# Cardiac glycoside/aglycones inhibit HIV-1 gene expression by a mechanism requiring MEK1/2-ERK1/2 signaling

**DOI:** 10.1038/s41598-018-19298-x

**Published:** 2018-01-16

**Authors:** Raymond W. Wong, Clifford A. Lingwood, Mario A. Ostrowski, Tyler Cabral, Alan Cochrane

**Affiliations:** 1 Department of Laboratory Medicine and Pathobiology, University of Toronto, Toronto, ON M5S1A8 Canada; 2 Division of Molecular Structure and Function, Hospital for Sick Children, Toronto, ON M5G1X8 Canada; 3 Department of Biochemistry, University of Toronto, Toronto, Ontario M5S1A8 Canada; 4 Keenan Research Centre for Biomedical Science of St. Michael’s Hospital Toronto, Toronto, ON M5B1W8 Canada; 5 Department of Medicine, University of Toronto, Toronto, Ontario M5S1A8 Canada; 6 Department of Immunology, University of Toronto, Toronto, ON M5S1A8 Canada; 7 Institute of Medical Science, University of Toronto, Toronto, ON M5S1A8 Canada; 8 Department of Molecular Genetics, University of Toronto, Toronto, ON M5S1A8 Canada

**Keywords:** Kinases, Ion channel signalling, Small molecules, Antivirals, RNA splicing

## Abstract

The capacity of HIV-1 to develop resistance to current drugs calls for innovative strategies to control this infection. We aimed at developing novel inhibitors of HIV-1 replication by targeting viral RNA processing—a stage dependent on conserved host processes. We previously reported that digoxin is a potent inhibitor of this stage. Herein, we identify 12 other cardiac glycoside/aglycones or cardiotonic steroids (CSs) that impede HIV growth in HIV-infected T cells from clinical patients at IC_50_s (1.1–1.3 nM) that are 2–26 times below concentrations used in patients with heart conditions. We subsequently demonstrate that CSs inhibit HIV-1 gene expression in part through modulation of MEK1/2-ERK1/2 signaling via interaction with the Na^+^/K^+^-ATPase, independent of alterations in intracellular Ca^2+^. Supporting this hypothesis, depletion of the Na^+^/K^+^-ATPase or addition of a MEK1/2-ERK1/2 activator also impairs HIV-1 gene expression. Similar to digoxin, all CSs tested induce oversplicing of HIV-1 RNAs, reducing unspliced (Gag) and singly spliced RNAs (Env/p14-Tat) encoding essential HIV-1 structural/regulatory proteins. Furthermore, all CSs cause nuclear retention of genomic/unspliced RNAs, supporting viral RNA processing as the underlying mechanism for their disruption of HIV-1 replication. These findings call for further *in vivo* validation and supports the targeting of cellular processes to control HIV-1 infection.

## Introduction

In the absence of an effective vaccine to prevent human immunodeficiency virus (HIV) infection, ~36.7 million people currently infected with HIV (2016) rely on the availability and efficacy of existing drug treatments^1,2^. Current antiretroviral therapies (ARTs) can prevent the onset of acquired immune deficiency syndrome (AIDS), but their efficacy is limited by toxicity, adherence to treatment, high cost, and transmission of drug-resistant viruses—representing over 7–24% of new infections in the United States and Europe^1,3–5^. Consequently, novel strategies reducing the chance of viral adaptation to drugs need to be explored^1^. In contrast to most existing ARTs which target rapidly evolving and mutation-prone viral enzymes and envelope (Env) interactions^1,6^, we explored the potential of altering HIV-1 RNA processing—a stage of the virus lifecycle not targeted by current ARTs and regulated by highly conserved cellular proteins and viral RNA elements (Supplementary Fig. S1)^7^. Recent studies have indicated that disrupting this stage of the virus lifecycle prevents the development of drug-resistant virus^8^.

In our initial evaluation of known splice modulator drug/compounds, we identified two FDA-approved drugs (chlorhexidine and digoxin) as inhibitors of HIV-1 replication which altered viral RNA processing^9–11^. The use of digoxin in the clinic and its effectiveness against HIV replication at *ex vivo* concentrations ~2–6 fold below those present in the serum of patients treated for heart conditions made its antiviral properties worthy of further exploration^10,12^. However, it was unclear which responses elicited by CSs upon interaction with its receptor, the Na^+^/K^+^-ATPase (NKA)/Na^+^ pump, are required for suppression of HIV-1 gene expression.

Various hypotheses have been suggested to explain the effect of CSs on cells. The “Na^+^-pump lag” hypothesis explains the positive inotropic action of CSs on the heart^13,14^ by proposing that CS binding and inhibition of NKA function results in increased intracellular Na^+^ concentration ([Na^+^]_i_), ultimately leading to a rise in free intracellular Ca^2+^ concentration ([Ca^2+^]_i_). The increased Ca^2+^ is subsequently stored in the sarco-/endoplasmic reticulum via a Ca^2+^-ATPase (SERCA), resulting in enhanced Ca^2+^ oscillations and stronger heart contractions that underly the therapeutic action of CSs. When Ca^2+^ levels exceed sarco-/endoplasmic reticulum storage capacity (due to excessive NKA inhibition), cardiac arrhythmias can occur in patients. Consequently, CSs have a limited therapeutic index (TI)^13,15,16^.

However, binding of the CS ouabain at low nanomolar concentrations also activates multiple signaling cascades with little to no inhibition of the Na^+^ pump in cardiac myocytes, renal epithelial cells, and other cell types^17–22^. Unlike the Na^+^-pump lag hypothesis, the NKA signalosome hypothesis implicates multiple α-subunit isoforms of the NKA in the relay of responses to the cell interior upon ouabain binding^14,23^. CS binding to the NKA leads to activation of Src kinase and tyrosine phosphorylation of multiple kinases^14,23^. For instance, CS-NKA interaction and Src activation recruits phospholipase C (PLC)-γ and inositol 1,4,5 trisphosphate (IP_3_) receptor (IP_3_R)^24,25^ (or the IP_3_R alone)^20,26^ to the N-terminal domain of the NKA α subunit, resulting in Ca^2+^ oscillations due to triggering of IP_3_R channels on the sarco-/endoplasmic reticulum to release Ca^2+^. Secondary messengers such as Ca^2+^ can consequently deliver diverse responses to the nucleus including regulation of host alternative RNA splicing^27,28^. In addition, CS binding to the NKA activates phosphatidylinositol-3-kinase (PI3K) which, in turn, increases activity of AKT and its downstream effectors, including nitric oxide synthase (eNOS) to produce reactive oxygen species (ROS) in cells^19,29,30^. Furthermore, Src activation by CSs can transactivate the epidermal growth factor receptor (EGFR), providing the necessary scaffold proteins to assemble and activate Ras, initiating classical mitogen-activated protein (MAP) kinase (MAPK) extracellular signal-regulated kinase (ERK) 1/2 signaling through the Raf-MAPK/ERK (MEK) 1/2 cascade^17,21,31,32^. Initiation of the Ras-Raf-MEK1/2-ERK1/2 pathway can deliver signals to the nucleus via ERK1/2 translocation or ROS release from mitochondria^19,30,33^. Finally, ouabain has been reported to activate other MAPKs, such as c-Jun N-terminal kinase (JNK) and p38, upon binding to the NKA^21,34^.

Since CSs can activate multiple signaling pathways, we hypothesized that CS inhibition of HIV-1 gene expression could be due to any one of these signaling cascades, potentially independent of the toxic/arrhythmogenic effects of Ca^2+^ flux. Defining the signaling mechanism involved could offer alternative strategies to control HIV-1 infection. We also examined other members of the CS family of FDA-approved drugs to identify modulators of viral RNA processing with improved inhibitor profiles^10^. In this report, we provide evidence that >3/4 of the CSs tested from the cardenolide/bufadienolide class have greater potency and *in vitro* and *ex vivo* TIs (or selectivity indices) compared to digoxin. While all compounds tested have similar effects on HIV-1 Gag/Env and viral RNA accumulation, they differ in their effects on the expression of HIV-1 regulatory factors (Rev/Tat) and phosphorylation of host serine/arginine-rich (SR) splicing factors (SRp20/SRSF3 and Tra2β). Furthermore, we observed that the inhibition of HIV-1 gene expression by CSs is independent of changes in Ca^2+^, PI3K-AKT, and JNK/p38 MAPKs but could be partially alleviated by inhibitors of MEK1/2-ERK1/2 signaling. Moreover, depletion of the NKA α subunit, proposed to promote Src kinase activation, and addition of the MEK1/2-ERK1/2 activator, anisomycin, also inhibit HIV-1 gene expression^23,35,36^. These results highlight a link between signaling upon CS binding and the inhibition of HIV-1 and, possibly, other viruses^37–40^. This study highlights the potential of small molecules to modulate viral RNA processing by altering the activity of cellular factors and offers alternative strategies for controlling HIV/AIDS.

## Results

### Cardenolide and bufadienolide classes of CSs are potent inhibitors of HIV-1 gene expression

**Figure 1:**
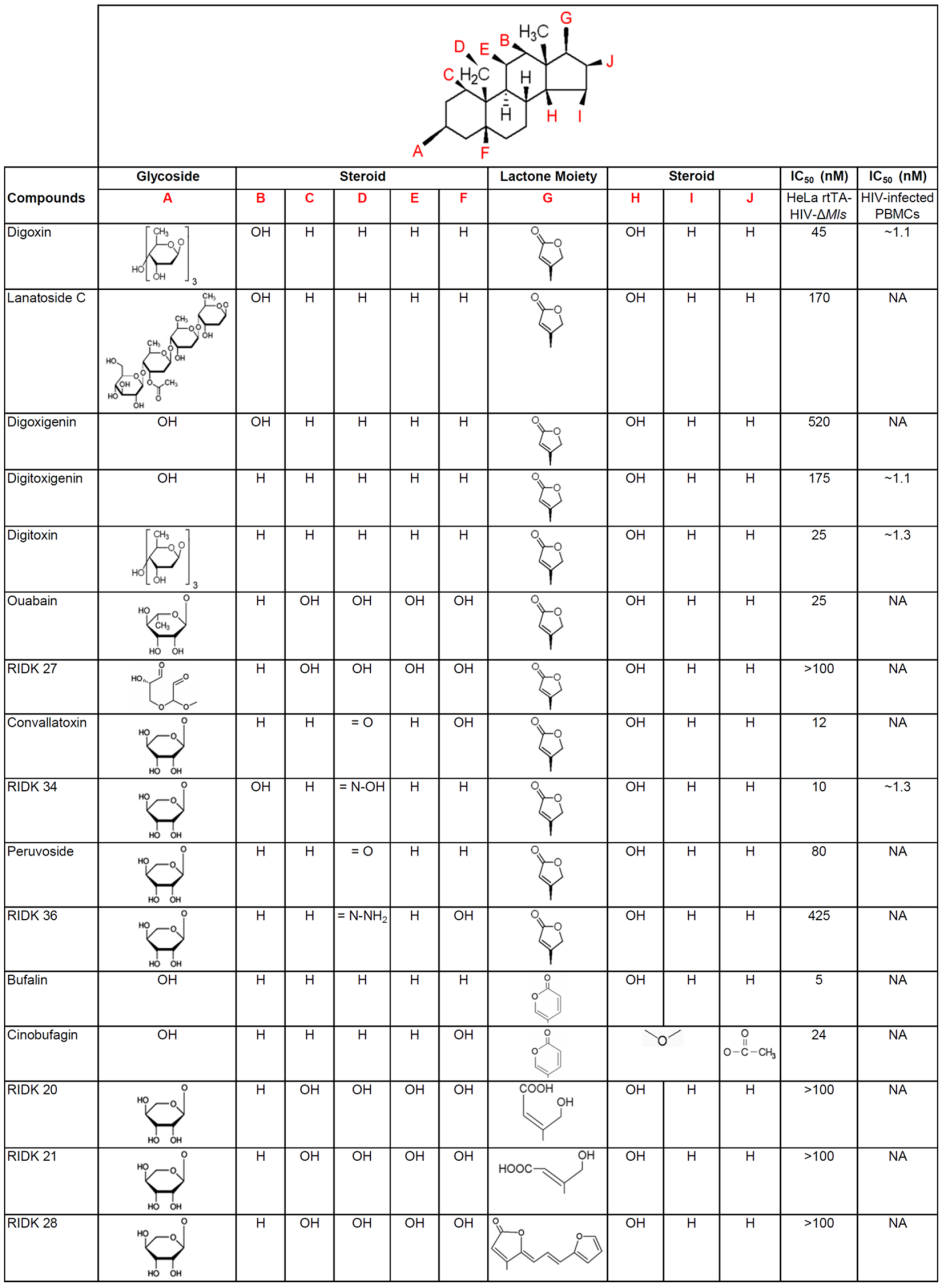
Chemical structure and inhibition data of cardiac glycoside/aglycones on HIV-1 gene expression. Chemical structure and dose-response data of CSs on HIV-1 (Gag) gene expression (IC_50_s) are summarized from Supplementary Figure S2 from HeLa rtTA-HIV-Δ*Mls* cells (except data was not shown from inactive compounds: RIDK-20, -21, -27, and -28) and Fig. 2 from HIV-infected PBMCs of patients. The steroid core and the position of various chemical substituents of CSs (red) are depicted (top). The different modifications of each CS (A–J) are listed below by chemical substituent. The presence and absence of a glycoside (a) distinguish between cardiac glycoside and aglycones, respectively. Differences in the lactone moiety from butyrolactone and α-pyrone (g) demarcate cardenolide and bufadienolide classes of CSs, respectively. The presence and absence of a hydroxyl group (−OH) at position 12 (b) describes “digoxin-like” and “digitoxin-like” CSs, respectively. Modifications of the steroid in (d–f) except for ouabain are specific to convallatoxin derivatives while changes in (h–j) are specific only to cinobufagin.

Members of the cardenolide and bufadienolide classes of CSs were evaluated in a HeLa rtTA-HIV-Δ*Mls* cell assay for their effect on HIV-1 (Gag) gene expression, summarized in Fig. 1 (dose-response curves provided in Supplementary Fig. S2), and their differences from digoxin described (IC_90_: 100 nM, IC_50_: 45 nM)^10^. All of the CSs tested suppressed HIV-1 gene expression without discernible changes in cell viability unless otherwise noted. For the cardenolide class (with highest similarity to digoxin, Fig. 1), addition of an extra glycoside on digoxin’s structure, lanatoside C (IC_90_: 370 nM), and aglycones (lacking glycosides) of digoxin and digitoxin: digoxigenin (IC_90_: N/A, IC_70_: 600 nM) and digitoxigenin (IC_90_: 500 nM), reduced anti-HIV-1 activity by ~4, 11, and 5 fold, respectively (Supplementary Fig. S2a–c), compared to digoxin (Fig. 1)^10^. However, unlike digoxigenin which elicited some cytotoxic effects (≥20%) at a concentration of 600 nM (CC_20_, Supplementary Fig. S2b), digitoxigenin had no toxicity at 1000 nM or >5.7 times above its IC_50_ (Supplementary Fig. S2c), suggesting that this compound, and perhaps other CSs (Fig. 1), may have better therapeutic profiles than digoxigenin/digoxin (see Discussion). Digitoxin (IC_90_: 45 nM, Fig. 1, Supplementary Fig. S2d), which differs from digoxin by the absence of a C-12 hydroxyl group on the steroid core, has an IC_50_ 2 fold lower for HIV-1 inhibition compared to digoxin (IC_90_: 100 nM). Ouabain, convallatoxin, and RIDK-34 also have improved anti-HIV-1 activity (IC_90_: 40, 24, and 25 nM, resp., Fig. 1, Supplementary Fig. S2e–g) relative to digoxin^12^. In contrast, RIDK-27, which resembles ouabain but contains a disrupted glycoside ring, had reduced activity (IC_50_: >100 nM, Fig. 1)^12^. Even minor differences in the steroid core from convallatoxin (IC_90_: 24 nM, Supplementary Fig. S2f, Fig. 1), such as peruvoside and RIDK-36 (IC_90_: 250 and ~1000 nM, switching –OH to –H and –CH=O to –CH=N-NH_2_, resp., Supplementary Fig. S2h–i), reduced potency 10- and 42-fold. The bufadienolide class (bufalin and cinobufagin, IC_90_: 15 and 40 nM, resp., Fig. 1, Supplementary Fig. S2j,k), which contains a 6-membered lactone moiety and have a higher average affinity for the NKA, are even more potent inhibitors of HIV-1 gene expression than cardenolides, with IC_50_’s 9 and 2.3 fold lower than digoxin^14^.

### CSs impede the growth of clinical strains of HIV from peripheral blood mononuclear cells (PBMCs) of HIV-infected patients

**Figure 2:**
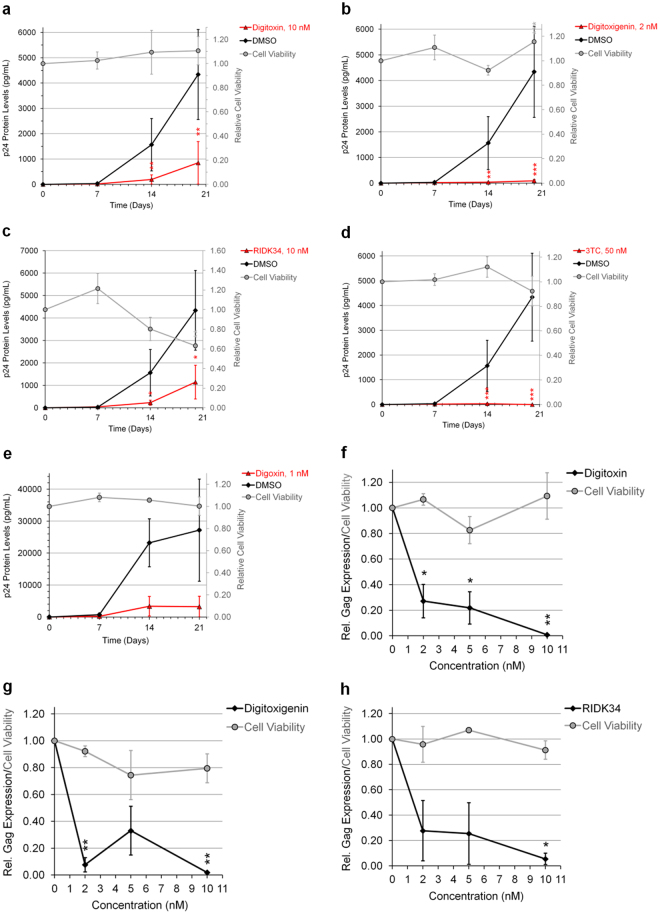
CSs inhibit the growth of clinical strains of HIV isolated from HIV infected patients. PBMCs from HIV infected patients were depleted of CD8^+^ T cells (Supplementary Fig. S3), activated by anti-CD3 and anti-CD28 antibodies, and treated 20 d with indicated concentrations of drug/compound. HIV particle formation was quantitated by p24^CA^ ELISA of cell supernatants and viability of cells were assayed by XTT. Data are displayed relative to DMSO (0 nM). (**a**–**e**) CSs potently impair the outgrowth of HIV from HIV-infected PBMCs (n = 3, mean, s.e.m.). Graphs of viral growth (p24 protein levels) and cell viability (gray circles, adjacent y-axis) of cells treated with drug/compounds (red triangles) or DMSO (black circles) are shown. The drug 3TC and the CS digoxin (**d**–**e**) were provided for comparison. (**f**–**h**) CSs potently inhibit HIV replication in a dose-dependent manner (n = 3, mean, s.e.m.). Dose-response curves of CS effects on HIV replication (relative Gag expression, black circles) and cell viability (gray circles) were measured on day 14.

The most promising cardenolides were subsequently tested on clinical strains of HIV using PBMCs from treatment-naïve HIV-infected patients. PBMCs were depleted of CD8^+^/cytotoxic T lymphocytes (Supplementary Fig. S3), activated, and virus outgrowth monitored in the presence/absence of drug/compound (Fig. 2). In contrast to DMSO-treated cells, addition of digitoxin or digitoxigenin exhibited strong suppression of viral growth (Fig. 2a,b) in a dose-dependent manner (IC_90s_: 7.5 and 2 nM, resp., Day14, Fig. 2f,g) with no observable changes in cell viability relative to controls. These results are comparable to the suppression of viral replication by 3TC and digoxin (IC_90_: ~5 nM and 2 nM, resp., Fig. 2d,e)^10^. In contrast, although RIDK-34 effectively inhibited HIV replication, it also reduced cell viability by day 20 of culture (Fig. 2c and h), indicating some level of cytotoxicity.

### CSs block the expression of vital HIV-1 structural and regulatory proteins

**Figure 3:**
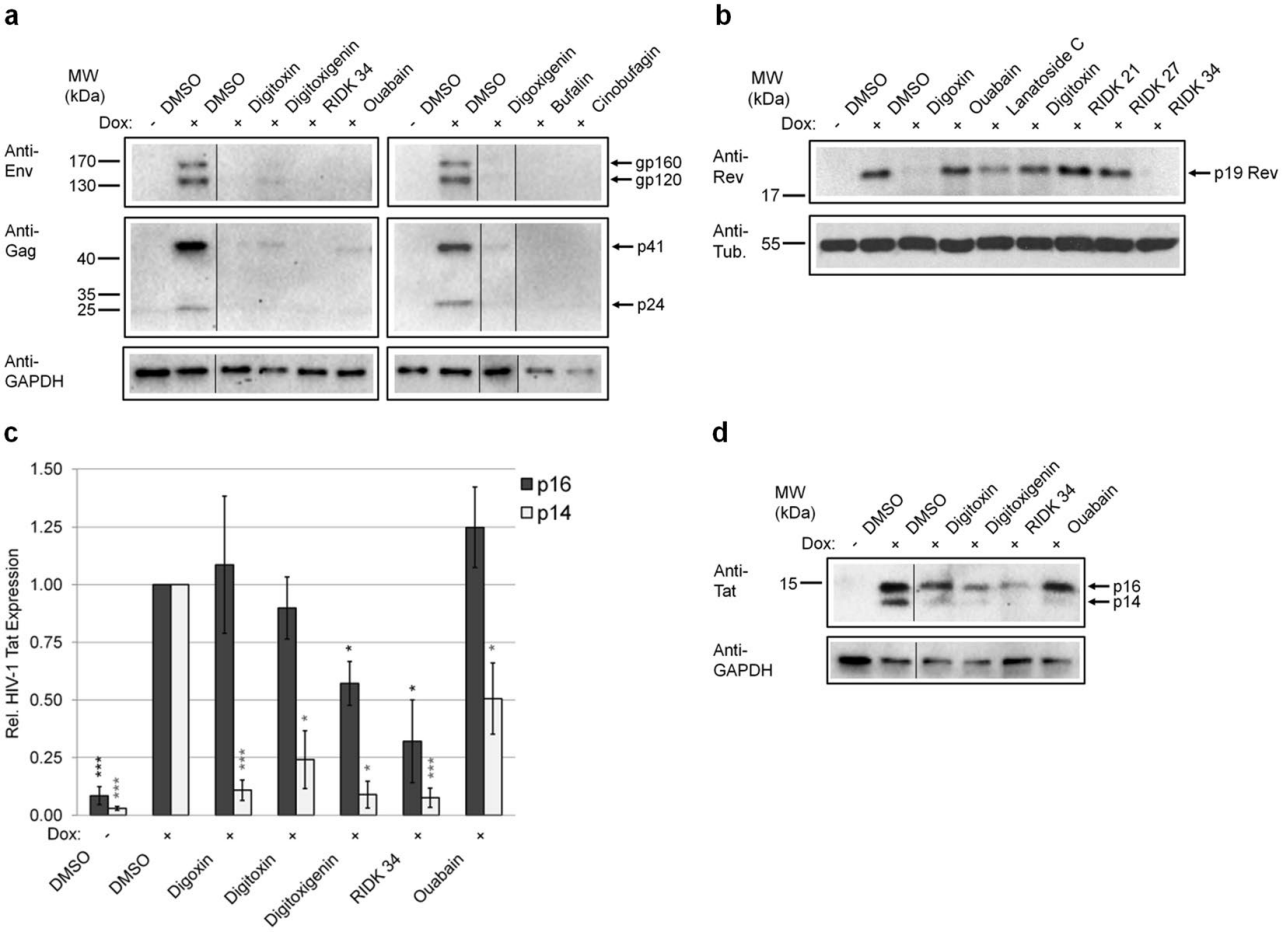
CSs reduce expression of essential HIV-1 structural and regulatory proteins. HeLa rtTA-HIV-Δ*Mls* cells were treated with ~IC_80_s of CSs (per Methods), 100 nM of RIDK-21 or -27, or DMSO for 4 h prior to Dox induction for 20 h as indicated. Cell extracts were analyzed by immunoblot of (**a**) HIV-1 structural proteins: Env (gp160/gp120) and Gag (p41/p24), and (**b**–**d**) viral regulatory factors: Rev (p19, **b**) and Tat (p16/p14, **c**,**d**). (**d**) Graph quantitating Tat expression. Results from (**a**–**d**) are from n ≥ 3, mean, s.e.m. GAPDH/tubulin served as internal loading control and for normalization of these data. (**a** and **d**) Lanes were cropped and assembled from the same blot per box from Supplementary Figure S4a,b and c, respectively.

To determine whether there were differences in how these compounds inhibited HIV-1 replication, we examined the effect of CSs on the expression of essential HIV-1 proteins in HeLa rtTA-HIV-Δ*Mls* cells (Fig. 3). All CSs tested significantly reduced the expression of Env polyprotein, gp160, and its processed product, gp120 (Fig. 3a). Consistent with dose-response curves (Supplementary Fig. S2), these drug/compounds also strongly decrease the levels of HIV-1 Gag polyprotein (p55) and its proteolytic processing intermediates, matrix-capsid (MA-CA, p41) and CA (p24) proteins, in cells relative to DMSO [+doxycycline (Dox)] control (Fig. 3a).

In our previous study, digoxin was shown to reduce expression of key HIV-1 regulatory factors, Rev and p14 Tat, without altering the early-expressed p16 Tat isoform (see Supplementary Fig. S1 on Tat isoforms)^10^. Rev functions by facilitating the export of incompletely-spliced [unspliced (US) or singly spliced (SS)] HIV-1 RNAs from the nucleus to the cytoplasm during late phase viral expression while Tat activates viral transcription during both early and late phases^41,42^. In evaluating the effect of other CSs, we noted that “digoxin-like CSs” (digoxin/lanatoside C/RIDK-34), containing a hydroxyl group at C-12 of the steroid (Fig. 1), reduce Rev accumulation relative to control (+, Fig. 3b). In contrast, CSs (Fig. 1) lacking the C-12 hydroxyl group (“digitoxin-like CSs”: digitoxin/ouabain) or those without anti-HIV-1 activity (RIDK-21/27) had no effect on Rev accumulation (Fig. 3b). Conversely, all CSs drastically decrease expression of p14 Tat while most of them have limited effects on p16 Tat levels (Fig. 3c,d). The exceptions were digitoxigenin and RIDK-34 which reduced p16 Tat expression to 57% and 32% of control, respectively. The differential effect of some CSs on both Rev and/or p16 Tat expression suggests potential mechanistic differences in the action of these drugs.

### Inhibition of HIV-1 gene expression by CSs is associated with alterations in viral RNA processing

**Figure 4:**
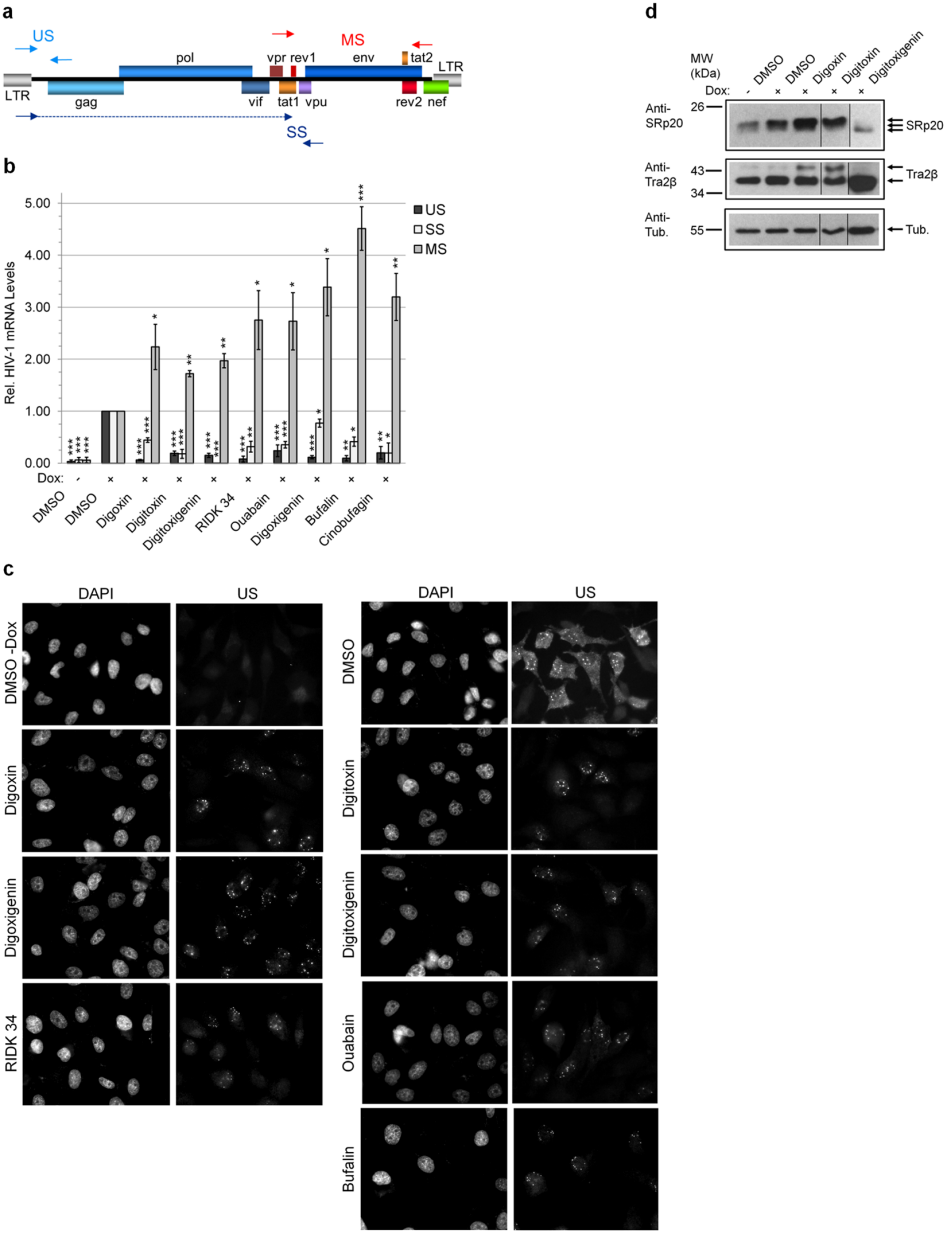
CSs suppress HIV-1 gene expression by altering viral RNA processing. HeLa rtTA-HIV-Δ*Mls* cells were treated with ~IC_80_s of CSs, RNAs extracted, quantitated by qRT-PCR or RT-PCR, and levels displayed relative to DMSO (+). (**a**,**b**) CSs induce oversplicing of HIV-1 RNAs (n ≥ 3, mean, s.e.m.). (**a**) Diagram of the primer positions (arrow heads) used for qRT-PCR. (**b**) Graph of the relative amount of US (black), SS (white), and MS (gray) HIV-1 RNAs in cells treated with various CSs. (**c**) Nuclear export of HIV-1 US RNAs in cells is altered by CSs (representative n ≥ 4). HIV-1 US RNAs were localized by FISH after treating HeLa rtTA-HIV(Gag-GFP) cells as described above but with ~IC_90_s of CSs. Nuclei were stained by DAPI and images captured at 630 × magnification. (**d**) CSs induce differential post-translational modification of SR proteins (representative n ≥ 3). SRp20, Tra2β, and tubulin were analyzed by immunoblot of HeLa rtTA-HIV-Δ*Mls* cells treated as described above except cells of this representative blot were treated 3 d with ~IC_50_s of CSs. (**g**) Lanes were cropped and assembled from the same gel/blot per box (Supplementary Fig. S4d).

To understand how CSs reduce expression of HIV-1 structural/regulatory proteins, RNA was isolated from HeLa rtTA-HIV-Δ*Mls* cells treated with CSs or DMSO and HIV-1 mRNA levels quantitated by qRT-PCR (Fig. 4a,b). All CSs tested decreased accumulation of incompletely-spliced HIV-1 RNAs: US RNA abundance being reduced to ~8 and 19% of control and SS RNA levels decreased to ~51 and 18% of control after treatment with digoxin- and digitoxin-like CSs, respectively, consistent with the reduced expression of Gag and Env/p14-Tat (Fig. 3a and c,d). Similar changes in HIV-1 RNA (and protein) accumulation were also observed upon addition of CSs (digoxin and digitoxin) to a CD4^+^ T-cell line (24ST1NLESG, Supplementary Fig. S5)^10^. However, the similar increases in MS mRNA abundance induced by all CSs does not explain their differential effect on Rev and p16 Tat protein accumulation (Fig. 3b–d), suggesting that differences between the compounds with respect to Rev likely occur at the level of translation or protein stability.

Additional experiments determined that all CSs tested induce a similar alteration in HIV-1 genomic RNA localization as detected by fluorescent *in situ* hybridization (FISH) of US RNAs in HeLa rtTA-HIV(Gag-GFP) cells (Fig. 4c). Control-treated cells had nuclear signal (with intensely labeled foci at putative sites of transcription) and strong cytoplasmic staining for US RNAs. In contrast, treatment with any CSs resulted in detection of US RNA signal almost exclusively within the nucleus (Fig. 4c), especially for digoxin-like CSs (digoxin/digoxigenin/RIDK-34), which reduce Rev accumulation (Fig. 3b), and bufalin. Some residual cytoplasmic staining for HIV-1 US RNAs was observed in cells treated with digitoxin-like CSs (digitoxin/digitoxigenin/ouabain, Fig. 4c) that do not affect Rev accumulation (Fig. 3b). These results indicate that, despite differences in their impact on Rev accumulation (and sometimes p16 Tat, Fig. 3b–d), all CSs block HIV-1 US RNA export to the cytoplasm.

### CSs induce post-translational modification of specific host splicing factors

The alterations in HIV-1 RNA accumulation by CSs (Fig. 4a–c) could be mediated by specific modification of cellular factors regulating RNA splicing, particularly SR proteins which generally enhance splicing and are regulated by their extent of phosphorylation^7,9^. Previously, we demonstrated that digoxin induces hyperphosphorylation of SRp20/SRSF3 (and modification of Tra2β). Furthermore, the drug-induced changes in HIV-1 RNA accumulation are comparable to those observed upon overexpression of SRp20^10^. Analysis of cell lysates determined that treatment with almost all CSs resulted in reduced mobility of SRp20 (Fig. 4d), comparable to digoxin-induced hyperphosphorylation^10^. Conversely, digitoxigenin treatment increased the mobility of SRp20 without affecting protein levels in a manner consistent with dephosphorylation^10^. In addition, CSs increased the abundance of a modified species of Tra2β while digitoxigenin and lanatoside C had no effect (Fig. 4d)^10^. Observed differences in these SR protein modifications and Rev/Tat protein accumulation (Fig. 3b–d) support the hypothesis that CSs differ in their mechanism of altering HIV-1 gene expression.

### The anti-HIV-1 activity of CSs requires interactions with the NKA

The effect of CSs on intracellular Ca^2+^ occurs via binding to the α subunit of the NKA^13,14,17–22^. We confirmed that the α1 but not the α2 isoform of the human NKA is expressed in all cell types used in this study (HeLa, SUPT1, and PBMCs, Supplementary Figs. S6a,b). Additionally, the α3 isoform is highly expressed in SUPT1s but detected only at background levels in PBMC and HeLa cells (relative to SUPT1s, Supplementary Fig. S6c). In support of the hypothesis that the antiviral activity of CSs is mediated through interactions with the NKA, we note the trend in anti-HIV-1 activity of ouabain, digitoxin, digoxin, and digoxigenin (Fig. 1) coincides with their reported binding affinities (1/K_d_) for NKA α subunits^10,43^. In addition, we found that alteration of the C-17 lactone group, which mediates CS binding to α subunits, results in reduced/no anti-HIV-1 activity of cardenolides (RIDK-20, -21, and -28, Fig. 1)^44^. These findings are further supported by a recent demonstration that overexpression of the mouse NKA α1 subunit, resistant to CS inhibition, blocks digoxin’s suppression of HIV-1 gene expression^12,45^.

**Figure 5:**
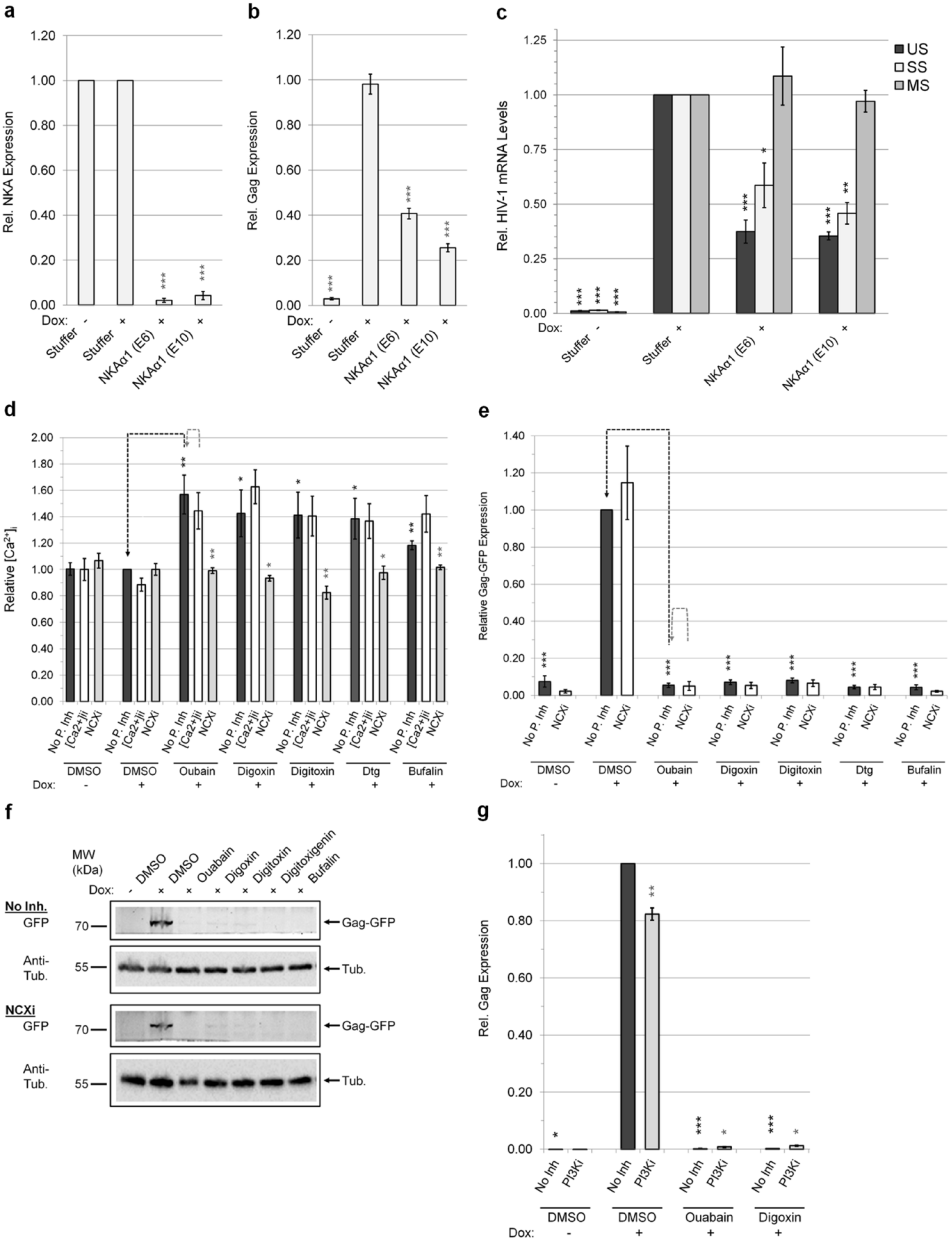
Effect of modulating NKA expression, [Ca^2+^]_i_, and PI3K-AKT on HIV-1 gene expression. Depletion of the NKA perturbs HIV-1 RNA processing (n ≥ 3, mean, s.e.m.). HeLa rtTA-HIV(Gag-GFP) cells were transduced with two shRNAs (E6/E10) to knockdown expression of the NKA α1 subunit and their effect on HIV-1 gene expression analyzed. After positive selection of cells with puromycin for 24–48 h, HIV-1 expression was Dox induced for 20 h, and cells harvested for analyses. Total mRNA (and protein) was isolated from cells, reverse transcribed, and analyzed by qRT-PCR and data normalized to β-actin (**a,c**). Results are shown relative to Stuffer (+). (**a**) Expression levels of NKA α1 mRNAs assayed by qRT-PCR. (**b**) HIV-1 Gag expression assayed by p24^CA^ ELISA of cell lysates (40 μg). (**c**) Accumulation of HIV-1 US (black), SS (white), and MS (gray) mRNAs quantified by qRT-PCR. (**d**–**g**) CSs suppress HIV-1 gene expression independent of intracellular Ca^2+^ flux/signaling and PI3K-AKT activation. HeLa rtTA-HIV(Gag-GFP) cells were pre-treated with either a chelator of [Ca^2+^]_i_ (5 µM BAPTA-AM, [Ca2+]ii), inhibitors of NCX Ca^2+^-entry (5 µM KB-R7943, NCXi) or PI3K (10 μM LY294002, PI3Ki), or untreated (DMSO, no pathway inhibitor) for ~2 h prior to treatment with ~IC_80_s of CSs or DMSO for 4 h, and Dox induced for ~20 h. The pathway used by a CS to inhibit HIV-1 expression was determined by monitoring for recovery of Gag-GFP expression by measuring GFP fluorescence in cell lysates (and initially by plate scans of live/fixed cells, Supplementary Fig. S7a and S8c). (**d**) Levels of [Ca^2+^]_i_ measured by Fura Red AM™ (n ≥ 4, mean, s.e.m.). (**e–f**) Quantification of Gag-GFP expression by reducing SDS-PAGE in lysates (35 μg) of cells which were pre-treated with/without KB-R7943 (n ≥ 3–4 mean, s.e.m.). Graph (**e**) and representative gel (**f**) of these results. Tubulin immunoblots serve as internal loading control and for normalization of this data. (**g**) Graph quantitating Gag expression by p24^CA^ ELISA in lysates of cells (35 μg) that were pre-treated with/without LY294002 (n ≥ 3, mean, s.e.m.). Statistical comparisons in (**a–g**) were performed as illustrated (black/gray dashed lines) or described in Methods.

As a further test of whether inhibition of the NKA could affect HIV-1 gene expression, we examined whether depletion of the NKA [using short hairpin RNAs (shRNAs) targeting the α1 subunit] could phenocopy the effect of CSs on HIV-1 protein and RNA accumulation. As detailed in Fig. 5a–c, shRNA depletion of the NKA α1 resulted in both a reduction in Gag expression and HIV-1 US/SS RNA abundance.

### CS inhibition of HIV-1 gene expression does not require changes in [Ca^2+^]_i_ or activation of PI3K-AKT signaling

To identify which intracellular pathways induced by CSs mediate their anti-HIV activity, we independently blocked Ca^2+^, PI3K, or the 3 MAPK signals to determine if any pathway inhibitor(s) could restore HIV-1 gene expression (as measured by recovery of Gag-GFP expression) in the presence of CSs. HeLa rtTA-HIV(Gag-GFP) cells were treated as described above except pre-treated with/without a pathway inhibitor prior to addition of CS or DMSO and induction of HIV-1 expression by Dox. To examine the role of Ca^2+^ oscillations (ionic/signaling), we used an intracellular Ca^2+^ chelator (BAPTA-AM) or an inhibitor of Ca^2+^ influx via the Na^+^/Ca^2+^ exchanger (NCX, KB-R7943)^15,34,46^. Consistent with functional NCXs being expressed in HeLa cells as reported^47^, all CSs triggered a significant rise in [Ca^2+^]_i_, which was blocked by addition of KB-R7943 but not by BAPTA-AM at concentrations with little/no effect on cell density (Fig. 5d, Supplementary Fig. S7a)^13,14,20^. However, neither of these Ca^2+^ blockers rescued HIV-1 Gag-GFP expression in the presence of CSs (Fig. 5e,f, Supplementary Fig. S7b). This data indicates that CS inhibition of HIV-1 gene expression is independent of increased cytoplasmic Ca^2+^ levels responsible for the toxicity of these drugs in people.

To test whether PI3K-AKT activation could affect HIV-1 gene expression within our system, we activated this pathway using either EGF addition or AKT overexpression. As shown in Supplementary Fig. S8a, addition of EGF, which activates PI3K via binding to EGFR, marginally enhances HIV-1 gene expression in HeLa rtTA-HIV(Gag-GFP) cells. Similarly, overexpression of the PI3K substrate AKT [wild-type (WT)/constitutively-active/inactive] in HeLa rtTA-HIV-Δ*Mls* cells resulted in a small increase in HIV-1 gene expression (Supplementary Fig. S8b). To test whether CSs inhibition of HIV-1 gene expression required the PI3K pathway, the effect of a specific PI3K inhibitor, LY294002, on HIV-1 gene expression was assessed in the presence or absence of CSs (Fig. 5g, Supplementary Fig. S8c,d). Although LY294002 reduced EGF-induced enhancement of HIV-1 Gag expression, it did not reverse the inhibition of HIV-1 Gag-GFP expression by CSs compared to controls (no pathway inhibitor and CS, Fig. 5g, Supplementary Fig. S8c).

### CS suppression of HIV-1 gene expression involves activation of the MEK1/2-ERK1/2 pathway

**Figure 6:**
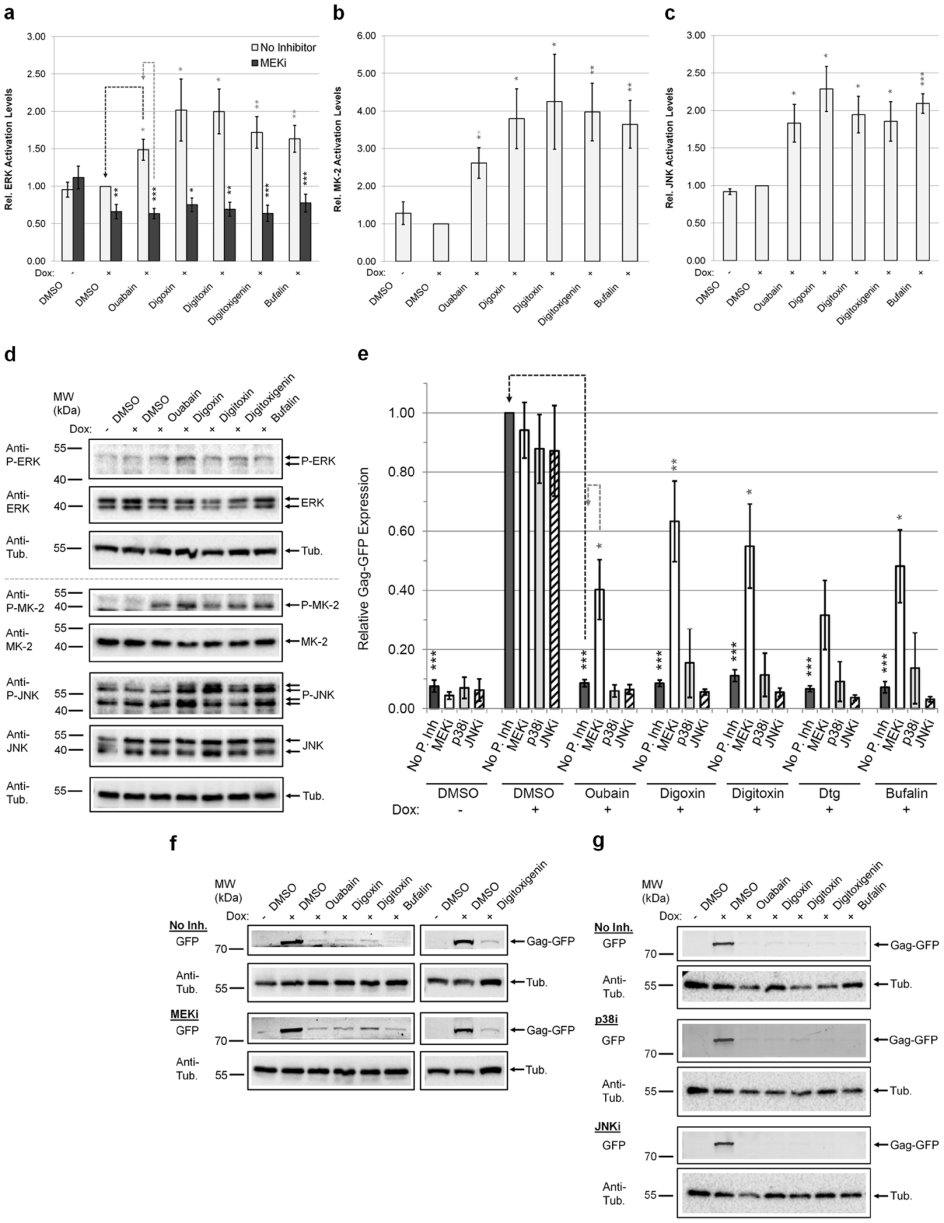
CSs control HIV-1 gene expression through intracellular signaling. HeLa rtTA-HIV(Gag-GFP) cells were pre-treated with/without pathway inhibitor overnight (~15 h) and treated with ~IC_80_s of CSs. All results were displayed relative to DMSO (+) with pre-treatment with no pathway inhibitor. (**a**–**c**) ERK1/2, MK-2, and JNK1/2/3 are activated upon treatment of cells with CSs (n ≥ 4–6, 3–4, and 3–6, resp., mean, s.e.m.). Graphs quantifying the activation level (phospho/total protein) of each MAP/MAPK by western blot. In (**a**), the results of pre-treating cells with a MEK1/2 inhibitor (12 μM U0126, MEKi) on ERK1/2 activation is also shown and a representative immunoblot is provided in Supplementary Figure S9c. (**d**) Representative immunoblots of MAP/MAPK activation levels from (**a**–**c**). (**e**–**g**) MEK1/2 activation may be involved in CS inhibition of HIV-1 gene expression. The signaling pathway(s) used by a CS to inhibit HIV-1 expression was determined by detecting Gag-GFP fluorescence in cell lysates (~35 μg) by reducing SDS-PAGE after pre-treatment of cells with a MEK1/2 (12 μM U0126, white), p38α/β/β2 (15 μM SB203580/p38i, gray), or JNK1/2/3 (1.25 μM SP600125/JNKi, hatched) inhibitor (n ≥ 5–7, 4–8, and 3–7, resp., mean, s.e.m.). Results were shown relative to DMSO (+). Tubulin immunoblots serve as internal loading control and for normalization of these data. (**e**) Graph and (**f**–**g**) representative gels of these results. Continuous lanes were cropped and assembled from 2 experiments for (**f**). Statistical comparisons were performed as illustrated (black/gray dashed lines) and described in Methods. Activity of each pathway inhibitor in cells was verified in (**a**) and Supplementary Figures S9c–g. Concentration of pathway inhibitor and CSs applied were predetermined to have little/no impact on total cell density (Supplementary Fig. S9h).

Consistent with reported literature, treatment of HeLa rtTA-HIV(Gag-GFP) cells with CSs activated ERK1/2 (MEK1/2’s target), MAPKAPK-2 (MK-2, p38’s target substrate), p38 (Supplementary Fig. S9a,b), and JNK1/2/3 relative to DMSO (+) controls (Fig. 6a–d)^21,22,34^. To determine whether any MAPK signaling pathway(s) is required for CS inhibition of HIV-1 gene expression, cells were pre-treated with inhibitors of MEK1/2 activity (U0126), p38α/β/β2 MAPK activity (SB203580), or JNK1/2/3 activation (SP600125) and monitored for Gag-GFP expression in the presence/absence of a CS. Each inhibitor was confirmed to block CS-induced activation of a specific MAP/MAPK with little/no effect on cell viability (Fig. 6a, Supplementary Fig. S9c–g). However, only pre-treatment of cells with a MEK1/2 inhibitor (U0126) partially restored HIV-1 gene expression for all CSs tested, with the exception of digitoxigenin (Dtg), relative to controls (no pathway inhibitor and CS, Fig. 6e,f). In contrast, inhibition of the other MAPKs (p38 and JNK) had no effect on HIV-1 Gag expression (Fig. 6e and g). To confirm this observation, the effect of Selumetinib/AZD6244, another specific inhibitor of MEK1/2 with distinct chemical structure, higher affinity (nM), and even lower selectivity entropy was examined^48,49^. Pre-treatment of cells with Selumetinib and subsequent addition of CSs (ouabain or digoxin) resulted in a response similar to that of U0126, blocking ERK activation and partially rescuing HIV-1 Gag expression (Supplementary Fig. S10a–c). The possibility that Ras-Raf-MEK1/2 or PI3K-AKT response could be attributed to a secondary effect through ROS (or vice-versa) and subsequent endo/exocytosis of the NKA is unlikely given that we found no noticeable change in the levels of ROS or NKA upon treatment with CSs for 24 h (Supplementary Fig. S11a–c)^19,22,50^. These data indicate that most CSs tested require, in part, signaling of the Raf-MEK1/2-ERK1/2 pathway to inhibit HIV-1 gene expression with little/no influence from activation of Ca^2+^ flux, PI3K-AKT, or p38/JNK MAPKs.

### Activation of the MEK1/2-ERK1/2 pathway by anisomycin suppresses HIV-1 gene expression

**Figure 7:**
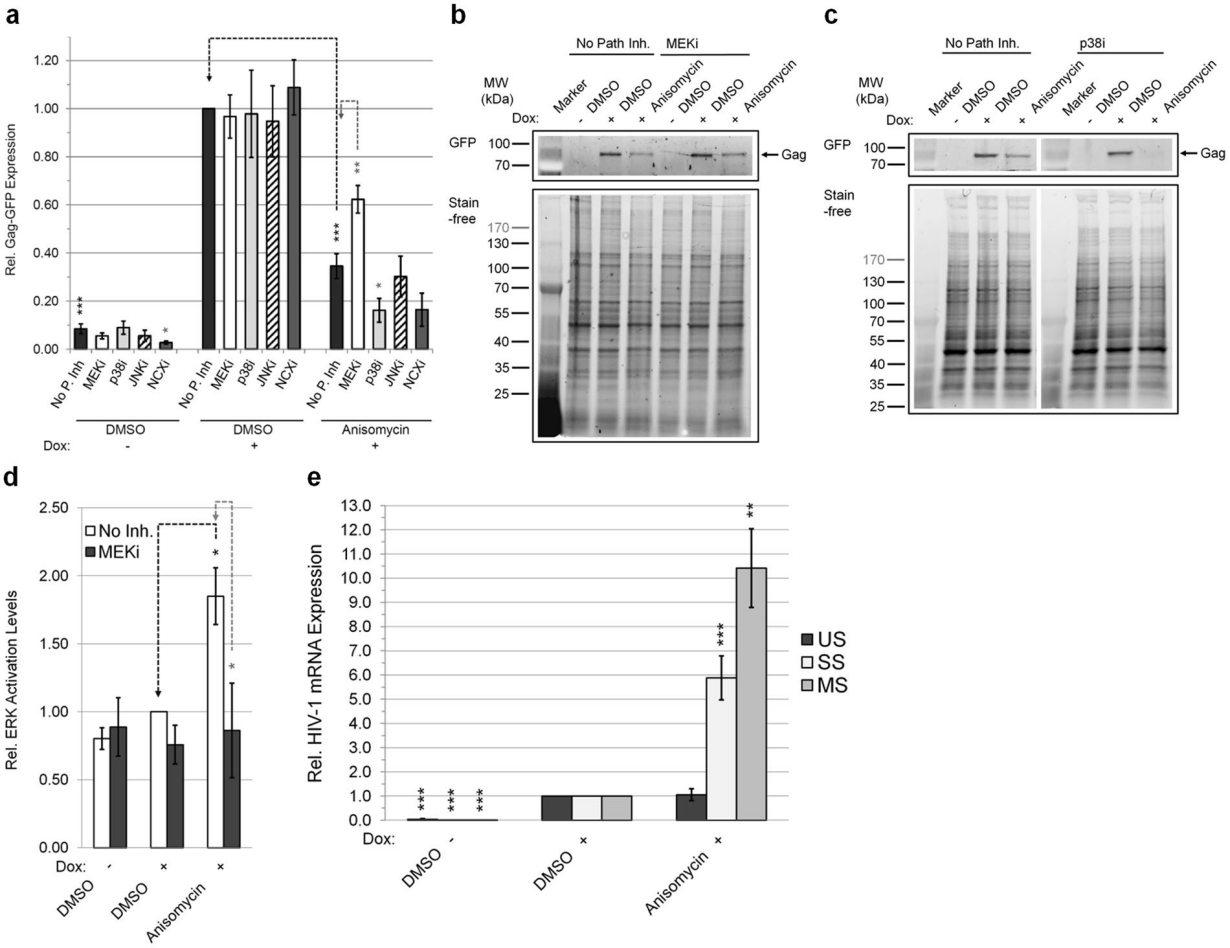
Activation of the MEK1/2-ERK1/2 signaling pathway suppresses HIV-1 gene expression. HeLa rtTA-HIV(Gag-GFP) cells were pre-treated overnight (~15 h) with/without an inhibitor of MEK1/2 (12 μM U0126, MEKi, white), p38α/β/β2 (15 μM SB203580, p38i, light gray), JNK1/2/3 (1.25 μM SP600125, JNKi, hatched), or NCX (5 μM KB-R7943, NCXi, gray) and treated with a MEK1/2-ERK1/2 activator, anisomycin, to isolate the pathway signal as described (and run in parallel for verification of inhibitor activity) in Figures 6 and 5d–f. Cells were monitored for HIV-1 gene expression by detecting Gag-GFP fluorescence in cell lysates (35 μg) resolved on reducing SDS-PAGE, levels of ERK activation by immunoblotting of phospho- and total-ERK from cell lysates, and extent of viral RNA expression by qRT-PCR of mRNAs extracted. Results were displayed relative to DMSO (+) that were pre-treated with no pathway inhibitor. Statistical comparisons were performed as illustrated (black/gray dashed lines) and described in Methods. (**a**) Graph of HIV-1 Gag-GFP expression in treated cells (n ≥ 5, mean, s.e.m.) and (**b**,**c** and Supplementary Fig. S12c) representative gels of these results. (**d**) Graph of ERK activation levels in the presence/absence of MEKi and anisomycin (n ≥ 5, mean, s.e.m.). Representative immunoblot of (**d**) is shown in Supplementary Figure S12a. Gels in (**b**,**c**) were run simultaneously and assembled from the same experiment. Stain-free™ total protein staining serves as internal loading control and for normalization of data in (**a**–**d**). (**e**) Graph of the accumulation of US, SS, and MS HIV-1 RNAs in anisomycin treated cells (n ≥ 3, mean, s.e.m.). RNAs were quantified by qRT-PCR as described in Figures 4a,b. Concentrations of MEKi and anisomycin applied in these experiments were predetermined to have little/no impact on total cell density (Supplementary Fig. S12b).

Given the possible role of MEK1/2-ERK1/2 signal activation in CSs modulation of HIV-1 gene expression, we explored whether anisomycin, a known activator of this pathway, could elicit a similar effect^35,36^. Addition of anisomycin to HeLa rtTA-HIV(Gag-GFP) cells inhibited HIV-1 Gag expression (Fig. 7a–c, Supplementary Fig. S10a and c), activated ERK1/2 **(**Supplementary Fig. S10b,c, Fig. 7d, Supplementary Fig. S12a**)**, and altered viral RNA accumulation (Fig. 7e**)**. Furthermore, as with CSs, inhibition of HIV-1 Gag expression by anisomycin was partially reversed upon pre-treatment with either U0126 (Fig. 7a,b) or Selumetinib (Supplementary Fig. S10a and c), but not inhibitors of p38/JNK MAPKs or NCX (Fig. 7a and c, Supplementary Fig. S12c). The concentration of anisomycin (220 nM) used had little to no effect on nascent protein synthesis or cell density in these assays (Supplementary Fig. S12b and d).

## Discussion

The possibility of repurposing drugs already used in humans as novel therapeutics for the control of HIV-1 infection is highly attractive. Our findings agree with a recent study which identified multiple CSs as inhibitors of HIV-1 expression^45,51^. Our study confirms that ≥ 5 of the reported hits and 7 additional CSs suppress HIV-1 gene expression in transformed cells [HeLa and CD4^+^ SUPT1s, Supplementary Figs S2 (or Fig. 1) and S5] and HIV replication in primary CD4^+^ PBMCs from HIV-infected clinical patients (Fig. 2) at low to single-digit nanomolar concentrations without cytotoxicity (summarized in Supplementary Table S1)^10,14,52–54^. The low concentration of CSs required to suppress viral replication in PBMCs (Fig. 1–2) suggests that these drugs could be used to treat HIV-1 at doses below those recommended for heart conditions^10^. Digitoxin (IC_50_: ~1.3 nM) requires 15–26 fold lower concentrations to inhibit viral replication in HIV-infected PBMCs than the recommended serum concentration in patients treated for heart conditions (20–34 nM), a substantial improvement over digoxin (which required a 2–6 fold lower dose)^10,12,55^. Although RIDK-34 and digitoxigenin are not in clinical use, they displayed at least ~1.5 fold better approximate *ex vivo* TI than their FDA-approved counterparts (Supplementary Table S1). In addition, ouabain (used in Europe), bufalin or cinobufagin (used in the traditional Chinese medicine Chansu), and convallatoxin, respectively, have 1.8, 9, 1.9, and 3.8 fold better anti-HIV-1 activity as well as 1.2, 9, 2.3, and 6.8 fold better *in vitro* TIs than digoxin in our cell based assays (Supplementary Table S1). These results indicate that many CSs that are not in clinical use may have better TIs for controlling HIV infection than for treating heart conditions and may be worth further investigation as HIV inhibitors.

**Figure 8:**
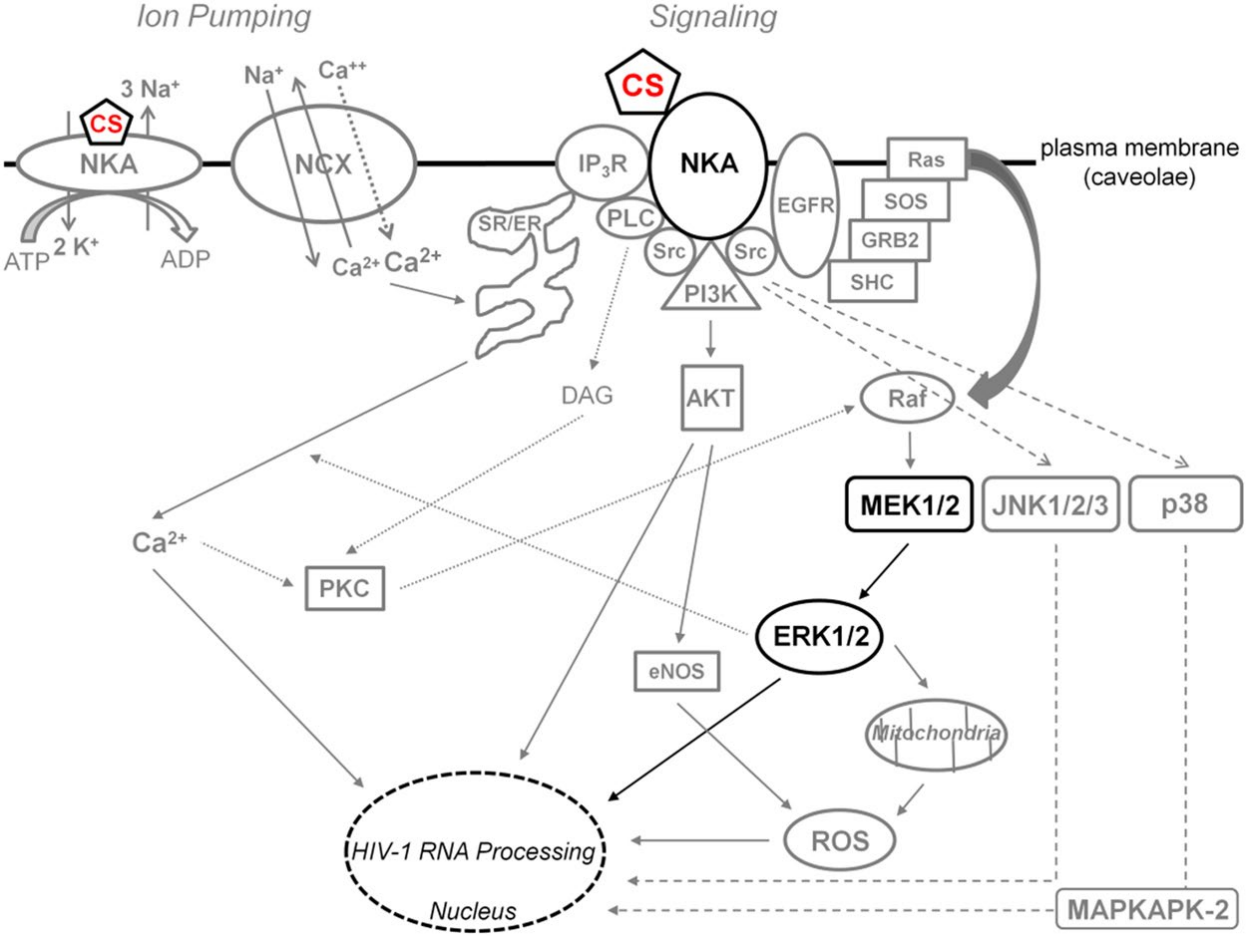
Model depicting the suggested signaling pathway modulated by CSs to inhibit HIV-1 gene expression. Although all CSs tested induce intracellular Ca^2+^ flux (left, gray) as well as activation of PLC-IP_3_R-Ca^2+^, PI3K-AKT, and JNK/p38 MAPK signaling via the NKA signalosome (right, gray), this study supports the hypothesis that CSs suppress HIV-1 gene expression in part through the MEK1/2-ERK1/2 signaling pathway (black), in a manner which is independent of NCX-mediated Ca^2+^ influx responsible for potential Ca^2+^ overload of the sarco-/endoplasmic reticulum (SR/ER) and triggering of toxicity/arrhythmias in patients.

Although very low doses of CSs were sufficient to inhibit HIV-1 replication in PBMCs, it should be noted that much higher concentrations were necessary in the assays using transformed cell lines (HeLa and SUPT1). While such differences might reflect the transformed nature of these cell lines (i.e. altered cell signaling), the enhanced activity of CSs in the context of primary cells could be attributed to the expansion of the infection. In addition to altering viral RNA processing (Fig. 4b,c, Supplementary Fig. S5e) and inducing intracellular signaling (Fig. 5–6 and 8, Supplementary Fig. S11–12), all CSs decreased p14 Tat (and digitoxigenin/RIDK-34 also affect p16 Tat, Fig. 3c,d) and, in the case of digoxin-like CSs, reduce Rev expression (Fig. 3b)^10^. While Tat is not required in the HIV-1 HeLa system used in this study, its essential role for viral transcription in the context of wild-type HIV would magnify the effect of CSs on viral growth (Fig. 2)^1,7,10^. The effect of CSs on Tat and Rev (Fig. 3b–d) and incompletely-spliced viral RNAs (oversplicing, Fig. 4b) would also lead to reduced availability of genomic HIV-1 RNAs for packaging into viral particles (Fig. 4c). Although CSs have also been reported to block HIV entry at high doses (4.2 µM), there is no data on whether similar effects are observed at the low nanomolar concentrations used here^56^. On the other hand, a recent report revealed that digoxin, can also inhibit HIV-1 infection by affecting sites of provirus integration^57^. Consequently, in the context of PBMCs (Fig. 2) and *in vivo*, where multiple rounds of viral replication would occur, synergy between effects at multiple different stages of HIV replication would greatly enhance the antiviral effects of CSs.

Consistent with data in previous reports, this study supports the hypothesis that CSs elicit their effect on HIV-1 gene expression (Fig. 1–4) through binding to the NKA (modeled in Fig. 8; Supplementary Fig. S6, S9, and S10)^13,14^. Although CS addition increased intracellular [Ca^2+^]_i_ (Fig. 5d), treatment with the NCX inhibitor KB-R7943 did not reverse the CS suppression of HIV-1 gene expression (Fig. 5e,f, Supplementary Fig. S7). These results indicate that modulation of intracellular Ca^2+^ is not required for the antiviral effect of CSs but this does not fully rule out a role for Ca^2+^ signaling *in vivo* or in prolonged cultures of HIV-infected PBMCs (2–3 weeks, Fig. 2) during which time an amplification of a CS effect could potentially occur. MAPK activation may require a rise in [Ca^2+^]_i_ and vice-versa since both pathways are linked via a positive feed-back cycle (Fig. 8)^58^. It is unlikely that the antiviral effect of CSs is due to inotropy or Ca^2+^ signaling amplified via a “plasmERosome” mechanism (described by Blaustein that requires NKA α2/3 subunits)^14,59,60^ since HeLa and PBMCs express no/limited amounts of NKA α2 or α3 isoforms required for this response (Supplementary Fig. S6)^61,62^. Supporting this hypothesis, the concentration of CS required to suppress HIV-1 gene expression in SUPT1s (Supplementary Fig. S5b), which does express the α3 isoform (Supplementary Fig. S6c), was higher, not lower than those used in the HeLa cell line that does not express this subunit (Fig. 1 or Supplementary Fig. S2; summarized in Supplementary Table S1)^10^. Our data is consistent with CS inhibition of HIV expression/replication being independent of drug-induced changes in intracellular Ca^2+^ (Fig. 5d–f and Supplementary Fig. S7b) that is responsible for the toxicity/arrhythmias of these drugs in patients^13,15,46^.

Although previous work has determined that CSs can have multiple effects on cells depending on the concentrations used^12,21,52,63,64^, CSs or anisomycin reduction of HIV-1 gene expression can be partially reversed only by inhibition of MEK1/2-ERK1/2 signaling (by either of two highly specific and distinct inhibitors, Fig. 6e,f, Supplementary Fig. S10) and is independent of PI3K-AKT (Fig. 5g, Supplementary Fig. S8b,c) or p38/JNK MAPKs activation (Fig. 6b–e, and g, Supplementary Fig. S9a,b and d–g)^48,49^. Likewise, depletion of the NKA α subunit (Fig. 5a–c), presumed to activate Src, inhibits HIV-1 gene expression. Each of these modulations (CSs, anisomycin, and shRNA depletion of NKA) altered HIV-1 RNA accumulation, with NKA depletion being the most similar to the response induced by CSs (Fig. 4b, 5c, and 7e). This data indicates that activation of the Src-EFGR-Ras-Raf-MEK1/2-ERK1/2 pathway contributes to the suppression of HIV-1 replication by CSs. Stimulation of the Ras-Raf-MEK1/2 pathway could relay signals to the nucleus via activation of ERK1/2, which has ≥ 200 substrates^65,66^. The successful use of CSs (modeled in Fig. 8) for the treatment of cancers in which the EGFR-Ras-Raf pathway is activated suggests that the anti-cancer and antiviral activity of this class of compounds may be very similar (Fig. 6a and d–f, Supplementary Fig. S9c and S10a–c)^14,52,54,67^.

Our data support the hypothesis that, at low nM concentrations, CSs modulate viral RNA processing to inhibit expression of an integrated HIV-1 provirus (Fig. 1–4, Supplementary Fig. S5). CSs reduce HIV-1 US and SS RNA accumulation (Fig. 4b, Supplementary Fig. S5e), resulting in decreased synthesis of vital HIV-1 structural (Gag/Env, Fig. 3a and also Fig. 2 and Supplementary Fig. S2 and S5b,c) and regulatory proteins (p14 Tat, and sometimes, p16 Tat and/or Rev; Fig. 3b–d, Supplementary Fig. S5d) necessary for new virion assembly, propagation, and infection. CSs also alter host alternative RNA splicing, affecting 1681 splicing events (~20.6% of 8,175 analyzed) in cells treated with digitoxin (q-value = 0, sepscore ≥ 1.0)^68^. However, although all CSs induced similar alterations in HIV-1 RNA accumulation, they differed in their effect on Rev expression. While digoxin-like CSs reduce Rev accumulation (necessary for facilitating US/SS RNA export), digitoxin-like compounds have little/no effect (Fig. 3b). Since the changes in Rev or p16 Tat accumulation (Fig. 3b–d) cannot be directly correlated with alterations in HIV-1 MS RNA abundance, the differences are likely due to effects at the level of translation or protein stability. Despite significant levels of Rev being expressed and retaining its ability to shuttle (see Supplementary Fig. S14), we observed little or no accumulation of viral US RNAs in the cytoplasm upon addition of digitoxin-like CSs (Fig. 4c). This observation suggests that the reduced accumulation of HIV-1 US RNAs in the cytoplasm is the result of decreased accessibility of viral RNAs for Rev interaction as a consequence of enhanced viral RNA splicing (Fig. 4b, Supplementary Fig. S5e). In support of this hypothesis, almost all CSs tested induce modification of SRp20/SRSF3 (similar to digoxin-induced hyperphosphorylation of this factor, the exception being digitoxigenin, Fig. 4d) whose overexpression results in similar alterations in HIV-1 RNA accumulation as described for digoxin^10^.

Together, our findings support the concept of using CSs as novel ARTs for controlling HIV infection and suggest that they might have a similar or better TI for treating HIV infection than heart conditions (Supplementary Table S1). The results also demonstrate that modulation of the NKA signalosome, particularly events involving MEK1/2-ERK1/2 activation, lead to suppression of HIV-1 gene expression (Fig. 8). Consequently, more refined modulation of the appropriate signaling pathways could serve as an alternative approach to control HIV-1 infection and bypass the cardiotoxic effects of CSs attributed to changes in intracellular Ca^2+^ (Fig. 8).

## Methods

### Dose response of drugs on HIV-1 gene expression

Drug/compounds were tested for effects on HIV-1 gene expression using inducible Tet-ON HIV-1 cell lines [HeLa rtTA-HIV-Δ*Mls* or rtTA-HIV(Gag-GFP)] containing a HIV-1 (*LAI*) provirus activatable by Dox or tetracycline transactivator (tTA)^69^. The rtTA-HIV-Δ*Mls* provirus was modified and used as previously reported^9–11,70^. The rtTA-HIV(Gag-GFP) provirus was generated by deletion of the *PR* and *RT* coding regions within *pol* and insertion of *GFP* to the 3′ of *gag*, creating GFP fused to the C-terminal of Gag. After 4 h of drug/compound treatment, HIV-1 gene expression was activated with Dox (2 μg/mL) or tTA (described below). Equal concentrations of DMSO were present in each experiment. After ~20 h, cells and media were harvested to monitor the effects of drug/compound treatments as described below. HIV-1 gene expression was quantified via p24^CA^ ELISA or monitoring Gag-GFP fluorescence in cells as described below. In parallel, cell viability of treatments were assessed by XTT assay (Sigma-Aldrich, #TOX2). For confirmatory tests, the CD4^+^ HIV-1 T-cell line, 24ST1NLESG, from J. Dougherty, was treated with drug/compounds and HIV-1 gene expression activated by 1.8 µM of phorbol 12-myristate 13-acetate (PMA) as previously described^10,11,71^.

The approximate concentrations of CSs used in experiments in HeLa rtTA-HIV-Δ*Mls* or rtTA-HIV(Gag-GFP) cells were as follows: ~IC_80_s: 90 nM digoxin, 40 nM digitoxin, 500 nM digitoxigenin, 20 nM RIDK-34, 36 nM ouabain, 800 nM digoxigenin, 11 nM bufalin, 40 nM cinobufagin, and 400 nM lanatoside C; ~IC_50_s: 45 nM, digoxin, 20 nM digitoxin, 25 nM lanatoside C, 25 nM ouabain, 12 nM RIDK-34, and 165 nM digitoxigenin; and ~IC_90_s: 95 nM digoxin, 45 nM digitoxin, 500 nM digitoxigenin, 25 nM RIDK-34, 40 nM ouabain, 800 nM digoxigenin, 15 nM bufalin, and 40 nM cinobufagin. Drug/compounds were purchased from Sigma (Digoxin, #D6003; Digitoxin, #D5878; Digoxigenin, #D9026; Digitoxigenin, #D9404; Ouabain, #O3125; Bufalin, #S961175; Cinobufagin, #C1272; and Anisomycin, #A9789) and derivatives of convallatoxin were synthesized from C. Lingwood’s lab of the Hospital for Sick Children (convallatoxin, peruvoside, RIDK-34, -36, -20, -21, -27, and -28). Nucleoside analog reverse-transcriptase inhibitor, Lamivudine (3TC), was obtained from the NIH AIDS Reagent Program (#8146). Recombinant human EGF was from Invitrogen (#PHG0314). Chemical structures were drawn in ChemSketch (ACD/Labs).

### Ethics statement

Experimental procedures were performed on PBMCs, obtained with written informed consent from volunteer blood donors, in accordance with relevant guidelines and regulations which were reviewed and approved by the University of Toronto Research Ethics Board.

### Assaying viral growth in HIV infected PBMCS

Human PBMCs were obtained for experiments from drug-naïve HIV-infected patients, depleted of CD8^+^ T cells using Dynabeads CD8 (Invitrogen, #111.47D), activated with anti-CD3 and anti-CD28 antibodies, and treated as previously described and above^10,11^. PBMCs were then seeded to 24-well plates in the presence/absence of indicated drug/compounds (0.5 mL final) which were pre-diluted in RPMI^+++^ in the same manner as described above. Every 3–4 days, ~0.25 mL of media was harvested for assays and replenished with ~0.25 mL of fresh medium with drug/compound and 20 U/mL of IL-2. HIV growth in cultures was monitored by p24^CA^ ELISA of cell supernatants harvested (detailed below) and the effect of compounds on cell viability were monitored by XTT assay.

### Analysis of the expression of HIV-1 and host cellular proteins

#### Immunological quantification of viral and host proteins

To monitor HIV-1 gene expression or replication, Gag release into cell culture supernatants were assayed by ELISA using a p24^CA^ antigen capture assay kit (AIDS & Cancer Virus Program, NCI-Frederick, Frederick, MD USA). Media harvested from HIV clinical isolates were diluted ~10 fold (or as necessary) prior to performing this assay. For analysis of HIV-1 and SR protein expression (and phosphorylation states with calf intestinal alkaline phosphatase treatments), cells were lysed and analyzed by western blot as previously outlined^10^. Phosphatase inhibitors (e.g. 10 mM sodium fluoride, 2 mM sodium orthovanadate) were added to solutions requiring phospho protein analyses. An anti-chicken NKA antibody (α6F, #a6F–c, Developmental Studies Hybridoma Bank, The University of Iowa, contributed by Douglas M. Fambrough, The Johns Hopkins University) was used as specified to detect NKA α1- and β1-subunits across species. Antibodies specific to respective phospho- and total-MAPK/MAP proteins for ERK1/2, JNK1/2/3, p38α/β/γ/δ, and MAPKAPK-2 were from Cell Signaling Technology (#9106, 9102, 9255, 9252, 9211, 9212, 3007, and 3042, resp.). Activation of MAPKs was determined by western blot quantitation and calculation of phospho/total protein levels. Clarity (Bio-Rad, #170-5060) or Western Lightning ECL reagent (Perkin-Elmer, #NEL101) were used for detection of signals from blots bound with HRP-conjugated antibodies and captured by either X-ray film or Bio-Rad ChemiDoc™ MP System as previously described^9^. Unsaturated protein bands in immunoblots/SDS-PAGEs were quantitated by ImageLab, normalized to internal loading controls (α-tubulin, GAPDH, or Stain-Free™ labeled total protein), and displayed relative to DMSO (+Dox). Stain-Free™ gels were casted and proteins were detected as described in 10% TGX-Stain-Free FastCast Acrylamide Kit from Bio-Rad (#161-0183). Images were exported as TIF files for assembly, rotation, and equal brightness/contrast adjustments in ImageJ or Microsoft Powerpoint. Some lanes were cropped and rearranged from the same blot/gel as indicated. In representative gel/blot sets, samples were electrophoresed from the same experiment as controls, resolved simultaneously on identically cast gel(s), transferred to same PVDF (by either wet electrophoretic or by Bio-Rad Trans-Blot® Turbo Transfer System), and detected at same time. Marked locations of molecular weight (MW) standards are shown on the left as a reference. DMSO (+) vs. (−) demonstrate successful activation of viral gene expression by Dox in all assays. *SUnSET analysis of total cellular protein synthesis*^72^. HeLa rtTA-HIV-Δ*Mls* cells were cultured in the presence/absence of compound and pathway inhibitor (if any) and Dox induced for ~24 h as already described, then treated with puromycin (10 μg/mL, Sigma-Aldrich, #P8833) for 30 min to label nascent proteins prior to harvest. As control, 10 µM of cycloheximide (Sigma-Aldrich, #C4849) was added to some cells prior to puromycin treatment. Cells were subsequently washed, whole cell lysates prepared, and proteins quantified by western blot using an anti-puromycin antibody (EMD Millipore, #anti-12D10) as described above. *Transfection of plasmid DNA into cells*. Transfection of pCMV6-HA-AKT-1 (WT, KM, or Myr) or CMV myc and with CMV tTA pA and CMV PLAP plasmids into HeLa rtTA-HIV-Δ*Mls* cells were performed by polyethylene imine (PEI) transfection as previously described^9,10^. Transfections in each experiment contained equal amounts of DNA and performed in Opti-MEM (Invitrogen, #31985070). An anti-HA antibody from Abcam (16B12, #ab130275) was for detection of HA-tagged proteins. *Depletion of NKA α subunits*. shRNAs in pLKO-TRC005 targeting the NKA α1 subunit (ATP1A1) were prepared by transfection of plasmid DNA [with pLKO-TRC005 with ATP1A1 (E6 or E10), PAX-2, and VSV-G] into 293T cells as described above. Resulting cell supernatants containing shRNAs packaged into pseudotyped lentiviruses were harvested for gene silencing experiments. ATP1A1 E6 and E10 shRNAs were targeted towards the NKA α1 subunit coding sequence (5′-GCCTTTCAGAACGCCTATTTG-3′) and 3′-UTR (5′-GTGTACTTCAGTCTTGGAGTT-3′), respectively. For experiments, HeLa rtTA-HIV(Gag-GFP) cells were seeded 1 d prior, transduced overnight with a NKA α1 shRNA supernatant with 8 µg/mL polybrene, selected with 1 µg/mL of puromycin for ~3 d, viral gene expression induced by Dox for ~20 h, and cells harvested for analyses.

### Determining the effect of drug/compounds on HIV-1 RNA processing and host gene expression

#### Quantitation of HIV-1 and cellular mRNA expression

RNA was extracted from cells, reverse transcribed, and resulting cDNAs subject to qRT-PCR quantification of HIV-1 mRNAs as previously described except reactions used iTaq™ Universal SYBR® Green Supermix (Bio-Rad, #172-5120) run on a Bio-Rad CFX384 Touch™ Real-Time PCR Detection System and analyzed with CFX Manager™^9^. NKA α1, 2, and 3 subunits were detected by published primers, amplified by iTaq using the same cycling temperature and times previously described for HIV-1 US/MS cDNAs except α1, 2, and 3 annealing temperatures were 61 °C^9,73^. All data was normalized to β-actin as internal loading control. Primers sequences for NKA α subunits were as follows: α1 forward (5′-AGTACACGGCAGTGATCTAAAGG -3′), α1 reverse (5′- CAGTCACAGCCACGATAGCAC -3′), α2 forward (5′-GGAGATGCAAGATGCCTTTCA-3′), α2 reverse (5′-GCTCATCCGTGTCGAATTTGA-3′), α3 forward (5′-GACCTCATTTGACAAGAGTTCGC-3′), and α3 reverse (5′-GGGCAGACTCAGACGCATC-3′). *Monitoring the subcellular localization of HIV-1 genomic RNA*. HeLa rtTA-HIV(Gag-GFP) cells were seeded on cover slips, treated with drug/compound, and processed for FISH to detect HIV-1 US RNAs using Stellaris™ probes (Biosearch Technologies) as previously detailed^10,11^.

### Analysis of cell signaling pathways

Using the same methods and conditions described for HeLa rtTA-HIV-Δ*Mls* cells, HeLa rtTA-HIV(Gag-GFP) cells were seeded in 48-/12-well plates and pretreated with/without pathway inhibitor prior to treatment with drug/compounds and Dox. Equal concentrations of DMSO were present in each experiment. HIV-1 gene expression was determined by detecting Gag-GFP fluorescence in cells by plate scans using a Typhoon Imager 9400 (Amersham Biosciences) or Typhoon FLA 9400 (GE) on ImageQuant, cell lysates by SDS-PAGE captured on ChemiDoc MP, or cell lysates by p24^CA^ ELISA. Before quantification, cells were washed with warm PBS and either scanned live (and harvested for protein analyses) or fixed in 3.7% paraformaldehyde/formaldehyde-PBS for subsequent analyses. Data from cell scans and SDS-PAGEs were quantitated using ImageJ and Image Lab software, respectively. To determine which pathway signal was used by a CS to inhibit HIV-1 gene expression, cells treated with CS were pre-treated with a specific pathway inhibitor and monitored for recovery of Gag-GFP expression. Pathway inhibitors were purchased from Sigma (BAPTA-AM, #A1076-25MG; U0126, #U120-1MG; SP600125, #S5567-10MG; SB203580, #S8307-1MG), Abcam (KB-R7943, #ab120284), BioShop (U0126, #U0U237.5), Millipore/Calbiochem (LY294002, #440204), or Selleckchem (Selumetinib/AZD6244, #S1008). In parallel, the cell viability of each pathway inhibitor and CS treatment combination were monitored by cell density staining of fixed cells with 2% methylene blue (BioBasic, #MB0342) in 50% ethanol and read at OD_664_ on a TECAN Infinite® 200 PRO or Biotek Cytation5. Pathways activated by CSs were monitored by western blot as described above. Changes in [Ca^2+^]_i_ and ROS, respectively, were monitored by loading live cells with Fura Red™ AM (Life Technologies, #F-3020) or CellROX® Deep Red Reagent (Life Technologies, #C10422) as outlined by the manufacturer and read as described for Gag-GFP above.

### Determining the *in vitro* and *ex vivo* TIs of CSs

Without median toxic dose (TD_50_) and half-maximal effective concentration (EC_50_) data available to calculate TIs (*in vivo*) from HIV patients treated with CSs, we determined the following TIs for each cell type treated with a CS (summarized in Supplementary Table S1): *in vitro* TIs from HeLa rtTA-HIV-Δ*Mls* cells using CC_20_/IC_50_ (instead of CC_50_/IC_50_), *in vitro* TIs from 24ST1NLESG cells using CC_50_/IC_50_, and *ex vivo* TIs from HIV-infected PBMCs using CC_50_/IC_50_ (at day 14 of culture approximated from available cell viability trends for CC_50_s of Supplementary Fig. S13 and inferred near maximal IC_50_s).

### Statistical analyses

Data was analyzed in Microsoft Excel and expressed as means ± standard error of the mean (s.e.m.). Differences between two groups of data, i.e. drug/compound treatment vs. control (DMSO + Dox/HIV/PMA/tTA) or shRNA vs. control (stuffer + Dox), were compared by Student’s *t*-test (two-tailed). In cell signaling experiment graphs, cells pre-treated with no pathway inhibitor and CS were compared to those with no pathway inhibitor and DMSO (+) as illustrated (black dashed lines) whereas cells pre-treated with a pathway inhibitor and CS within a treatment set were compared to those with no pathway inhibitor and the CS within the same set (gray dashed lines). Statistical significance in results are indicated on graphs for each p value as follows: *p < 0.05, **p < 0.01, and ***p < 0.001, unless otherwise noted.

## Electronic supplementary material

Supplementary Information
