# Author Correction: Cardiac glycoside/aglycones inhibit HIV-1 gene expression by a mechanism requiring MEK1/2-ERK1/2 signaling

**DOI:** 10.1038/s41598-019-54732-8

**Published:** 2019-12-04

**Authors:** Raymond W. Wong, Clifford A. Lingwood, Mario A. Ostrowski, Tyler Cabral, Alan Cochrane

**Affiliations:** 1 Department of Laboratory Medicine and Pathobiology, University of Toronto, Toronto, ON M5S1A8 Canada; 2 Division of Molecular Structure and Function, Hospital for Sick Children, Toronto, ON M5G1X8 Canada; 3 Department of Biochemistry, University of Toronto, Toronto, Ontario M5S1A8 Canada; 4 Keenan Research Centre for Biomedical Science of St. Michael’s Hospital Toronto, Toronto, ON M5B1W8 Canada; 5 Department of Medicine, University of Toronto, Toronto, Ontario M5S1A8 Canada; 6 Department of Immunology, University of Toronto, Toronto, ON M5S1A8 Canada; 7 Institute of Medical Science, University of Toronto, Toronto, ON M5S1A8 Canada; 8 Department of Molecular Genetics, University of Toronto, Toronto, ON M5S1A8 Canada

Correction to: *Scientific Reports* 10.1038/s41598-018-19298-x, published online 16 January 2018

The Acknowledgements section in this Article is incomplete.

“We thank M. Mylvaganam and J. La of C.A.L.’s lab for convallatoxin derivatives; S. Mujib for assistance in preparing HIV-infected PBMCs for culture; A. Y.Q. Mao for AKT-1 transfections; T. Hoque and S.-K. Whyte of R. Bendayan’s lab for BMVEC cDNAs and L. Ming for THP-1 cDNAs and NKA shRNA supernatants; A. Balachandran for phytohemagglutinin-leucoagglutinin activated PBMC aliquot; M. Haaland for assisting A.C. in Rev immunofluorescence microscopy with digitoxin/ouabain; human blood donors for PBMCs; and J. Baxter, S. Kidane, W. X. Cao, and A. T.Y. Chen for proofreading manuscript.”

should read:

“We thank M. Mylvaganam and J. La of C.A.L.’s lab for convallatoxin derivatives; S. Mujib for assistance in preparing HIV-infected PBMCs for culture; A. Y.Q. Mao for AKT-1 transfections; T. Hoque and S.-K. Whyte of R. Bendayan’s lab for BMVEC cDNAs and L. Ming for THP-1 cDNAs and NKA shRNA supernatants; A. Balachandran for phytohemagglutinin-leucoagglutinin activated PBMC aliquot; M. Haaland for assisting A.C. in Rev immunofluorescence microscopy with digitoxin/ouabain; human blood donors for PBMCs; and J. Baxter, S. Kidane, W. X. Cao, and A. T.Y. Chen for proofreading manuscript.

This work was supported by a Canadian Institutes of Health Research (CIHR) Operating Grant - Priority Announcement: HIV/AIDS Research Initiative (HOP-134065) to A.C. and CIHR Doctoral Award - Frederick Banting and Charles Best Canada Graduate Scholarship to R.W.W."

