# Supplementary material for: Cardiac glycoside/aglycones inhibit HIV-1 gene expression by a mechanism requiring MEK1/2-ERK1/2 signaling
Source: Sci Rep. 2018 Jan 16;8:850. doi: 10.1038/s41598-018-19298-x (PMC5770468; doi:10.1038/s41598-018-19298-x)
Supplement: Supplementary file 1 — Supplementary Information [file 41598_2018_19298_MOESM1_ESM.pdf]

Supplementary Information

Cardiac glycoside/aglycones inhibit HIV-1 gene expression by a mechanism requiring MEK1/2-ERK1/2 signaling

Raymond W. Wong[1], Clifford A. Lingwood[1,2,3], Mario A. Ostrowski[4,5,6,7], Tyler Cabral[8], and Alan Cochrane[7,8*]

[1]Department of Laboratory Medicine and Pathobiology, University of Toronto, Toronto, ON, Canada, M5S1A8. [2]Division of Molecular Structure and Function, Hospital for Sick Children, Toronto, ON, Canada, M5G1X8. [3]Department of Biochemistry, University of Toronto, Toronto, Ontario, M5S1A8, Canada. [4]Keenan Research Centre for Biomedical Science of St. Michael's Hospital Toronto, Toronto, ON, Canada, M5B1W8. [5]Department of Medicine, University of Toronto, Toronto, Ontario, M5S1A8, Canada. [6]Department of Immunology, University of Toronto, Toronto, ON, Canada, M5S1A8. [7]Institute of Medical Science, University of Toronto, Toronto, ON, Canada, M5S1A8. [8]Department of Molecular Genetics, University of Toronto, Toronto, ON, Canada, M5S1A8. *Correspondence and requests for materials should be addressed to A.C. (email: alan.cochrane@utoronto.ca)

<u>Supplementary Results:</u>

Supplementary Table S1

Supplementary Figures S1-14

Supplementary Table S1. Comparison of the *in vitro* (IVTI) and *ex vivo* therapeutic indices (EVTIs) of CSs and their impact on the expression of essential HIV-1 proteins.

HeLa rtTA-HIV-Δ*Mls* cells.

Compound:	Bufalin	Peruvo side	Convalla toxin	Cinobuf agin	Digitoxigen in	RIDK 34	Ouabain	Digitoxin	RIDK 36	Digoxin	Lanato side C	Digoxigen in
CC_{20} (nM)[a]:	125	>1000	82	~155	>1000	37.5	85	70	>1000	125	470	575
IC_{50} (nM):	5	80	12	24	175	10	25	25	425	45	170	520
IC_{50} rel. to digoxin (fold change)	9.0	0.6	3.8	1.9	0.3	4.5	1.8	1.8	0.1	1.0	0.3	0.1
IVTI (CC_{20}/IC_{50})[a]:	25.0	>12.5	6.8	6.5	>5.7	3.8	3.4	2.8	>2.4	2.8	2.8	1.1
IVTI rel. to digoxin (fold change)	9.0	>4.5	2.5	2.3	>2.1	1.4	1.2	1.0	>0.9	1.0	1.0	0.4
Effect on Env[b]	reduced			reduced	reduced	reduced	reduced	reduced		reduced		reduced
Effect on p14 Tat[b]					reduced	reduced	reduced	reduced		reduced		
Effect on p16 Tat[b]					reduced	reduced	unaffected	unaffected		unaffected		
Effect on Rev[b]					(unaffected)	reduced	unaffected	unaffected		reduced	reduced	(reduced)

24ST1NLESG cells.

Compound:	Digitoxin	Digoxin
CC_{50} (nM):	>300	>500
IC_{50} (nM):	50	110
IC_{50} rel. to digoxin (fold change)	2.2	1.0
IVTI (CC_{50}/IC_{50}):	>6	>5
IVTI rel. to digoxin (fold change)	≥1.2	1.0

HIV-infected PBMCs.

Compound:	Digitoxigen in	RIDK 34	Digitoxin	Digoxin
CC_{50} (nM):	>80	~100	68	>55
IC_{50} (nM):	~1.1	~1.3	~1.3	~1.1
IC_{50} rel. to digoxin (fold change)	1.0	0.9	0.9	1.0
EVTI (CC_{50}/IC_{50}):	>73	~77	52	>50
EVTI rel. to digoxin (fold change)	1.5	1.5	1.0	1.0

Notes: data for CC_{20}, CC_{50}, and IC_{50}s for each drug/compound were estimated from dose response curves in **Figures 1-2**, **Supplementary Table S1**, **Supplementary Figures S2, S5**, and **S13**, and Wong *et al.* (2013). *PLoS Pathog* **9**(3):e1003241.

">" means the estimate has not been accurately determined but is greater than the highest concentration tested.

"( )" indicates preliminary data that was not shown.

[a]CC_{20} instead of CC_{50} was estimated from dose response curves and used for calculation of IVTIs.

[b]Cells were treated at ~$IC_{90}$of a CS.

Supplementary Figure S1

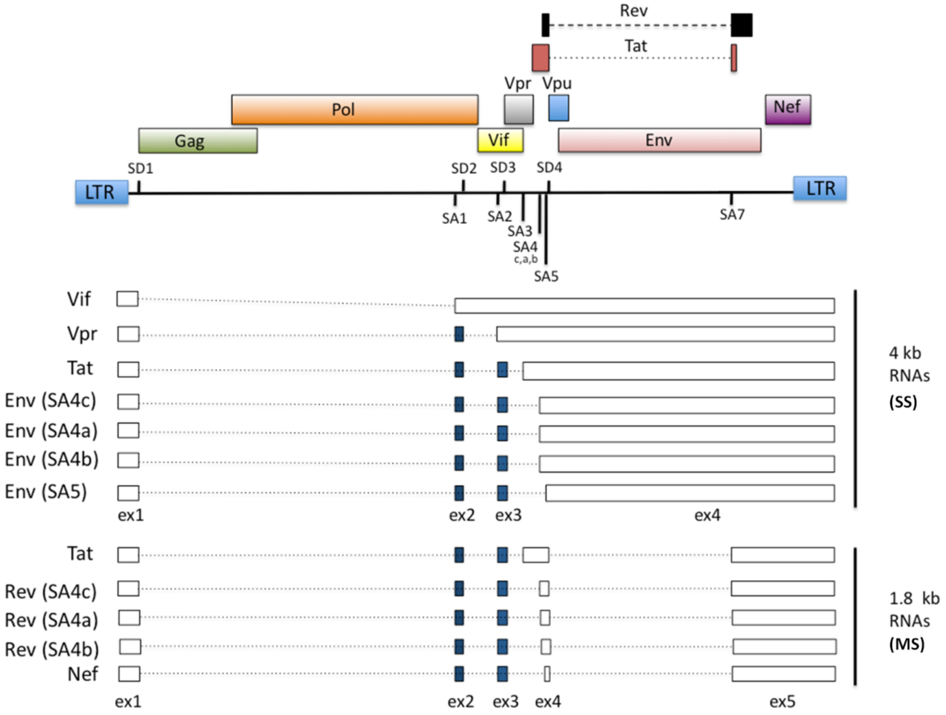

4 kb SS RNA species

vif	ex1-ex4(SA1)
vpr3	ex1-ex4(SA2)
vpr4	ex1-ex2-ex4(SA2)
tat5	ex1-ex4(SA3)
tat6	ex1-ex2-ex4(SA3)
tat7	ex1-ex3-ex4(SA3)
tat8	ex1-ex2-ex3-ex4(SA3)
env1	ex1-ex4(SA5)
env2	ex1-ex4(SA4b)
env3	ex1-ex4(SA4a)
env4	ex1-ex4(SA4c)
env5	ex1-ex2-ex4(SA5)
env6	ex1-ex2-ex4(SA4b)
env7	ex1-ex2-ex4(SA4a)
env8	ex1-ex3-ex4(SA5)
env9	ex1-ex2-ex4(SA4c)

1.8 kb MS RNA species

tat1	ex1-ex4(SA3)-ex5
tat2	ex1-ex2-ex4(SA3)-ex5
tat3	ex1-ex3-ex4(SA3)-ex5
tat4	ex1-ex2-ex3-ex4(SA3)-ex5
rev1	ex1-ex4(SA4b)-ex5
rev2	ex1-ex4(SA4a)-ex5
rev3	ex1-ex4(SA4c)-ex5
rev4	ex1-ex2-ex4(SA4b)-ex5
rev5	ex1-ex2-ex4(SA4a)-ex5
rev6	ex1-ex2-ex4(SA4c)-ex5
rev7	ex1-ex3-ex4(SA4b)-ex5
rev8	ex1-ex3-ex4(SA4a)-ex5
rev9	ex1-ex3-ex4(SA4c)-ex5
nef1	ex1-ex5
nef2	ex1-ex4 (SA5)-ex5
nef3	ex1-ex2-ex4(SA5)-ex5
nef4	ex1-ex3-ex4(SA5)-ex5
nef5	ex1-ex2-ex3-ex4(SA5)-ex5
vpr1	ex1-ex4(SA2)-ex5
vpr2	ex1-ex2-ex4(SA2)-ex5

Supplementary Figure S1. Pattern of HIV-1 mRNAs generated from splicing. Depicted is the organization of the HIV-1 proviral genome (top) indicating the position of multiple 5' splice donor sites (SD1-4) and 3' splice acceptor sites (SA1-7) used in the splicing of viral pre-mRNA. Below is an illustration of the alternatively spliced RNAs generated by processing HIV-1 genomic RNA (unspliced, US, middle). Indicated are the common (open boxes) and alternative exons (closed boxes) used in generating the singly spliced (SS, 4 kb) and multiply spliced (MS, 1.8 kb) viral RNAs. At the bottom is a list of the nomenclature used to describe the exon composition of each RNA generated from these two classes of HIV-1 RNAs. Note: there are two isoforms of Tat generated from these exons: p14 Tat from SS RNAs and p16 Tat from MS RNAs. The SS RNA generates a truncated form of Tat (p14) due to the presence of a termination codon immediately 3' of SD4, thereby translating it into a shorter isoform than MS RNAs.

Supplementary Figure S2

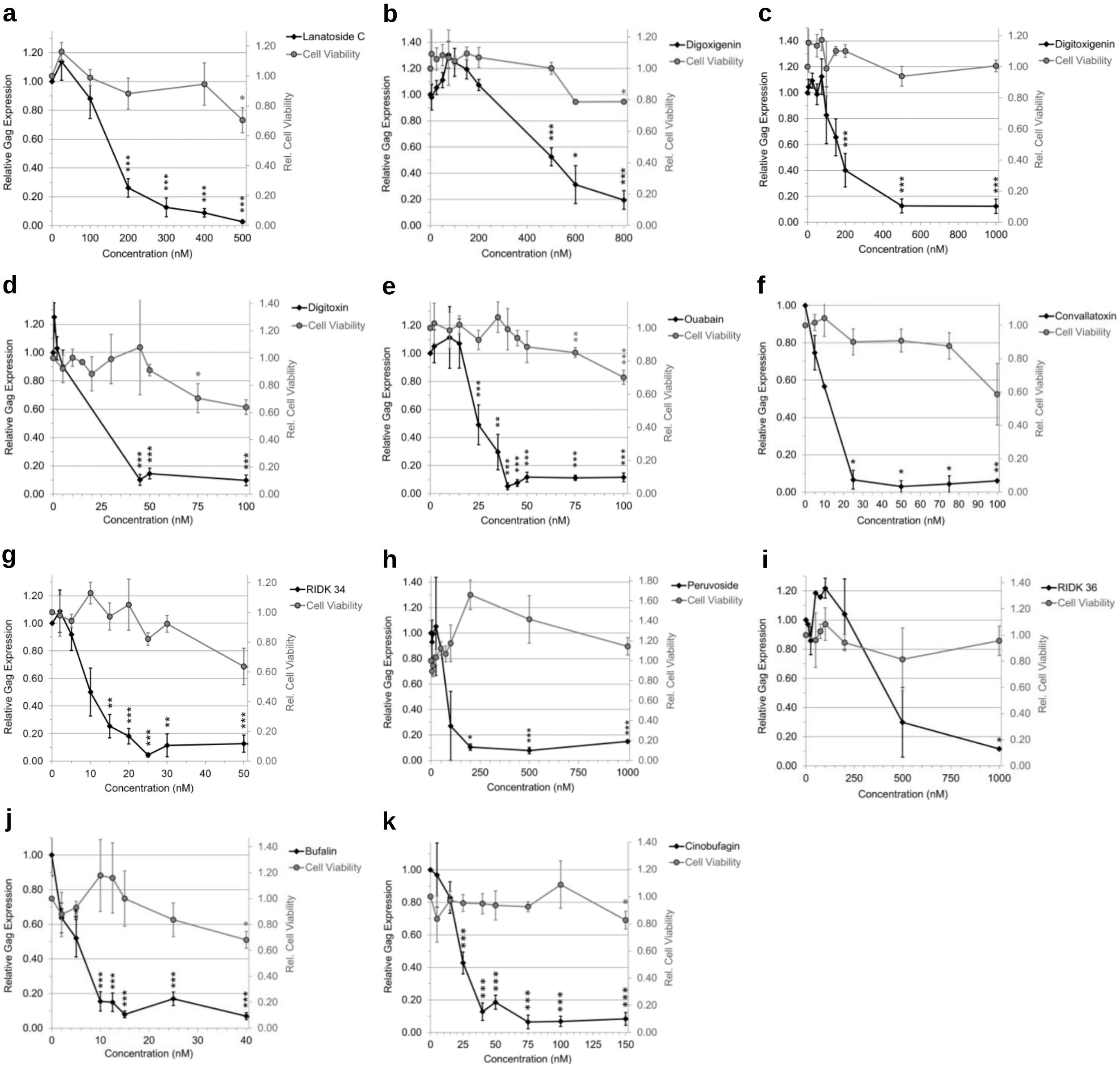

Supplementary Figure S2. CSs inhibit HIV-1 gene expression in a dose-dependent manner. HeLa rtTA-HIV-Δ*Mls* cells were treated with various cardenolides (**a-i**), convallatoxin and its derivatives (**f-i**), or bufadienolides (**j-k**) at concentrations indicated for 4 h prior to induction of viral gene expression with Dox for ~20 h. Dose response curves for each CS on HIV-1 gene expression were generated from $p24^{CA}$ ELISA of supernatants harvested from cells (black diamonds, y-axis, n ≥ 4, mean, s.e.m.) and, in parallel, their effects on cell viability assayed by XTT (gray circles, adjacent y-axis, n ≥ 4, mean, s.e.m.). Equal concentrations of DMSO solvent were present in each experiment. Peak Gag expression averaged ~1000 pg/mL in media harvested from induced cells. Data are displayed as a fraction relative to DMSO (+Dox) control (0 nM). Statistical analysis was performed per Methods. Results were compared to digoxin's published IC_{90} of 100 nM [Wong *et al.* (2013). *PLoS Pathog* **9**(3):e1003241] and were summarized in **Figure 1** and **Supplementary Table 1.**

Supplementary Figure S3

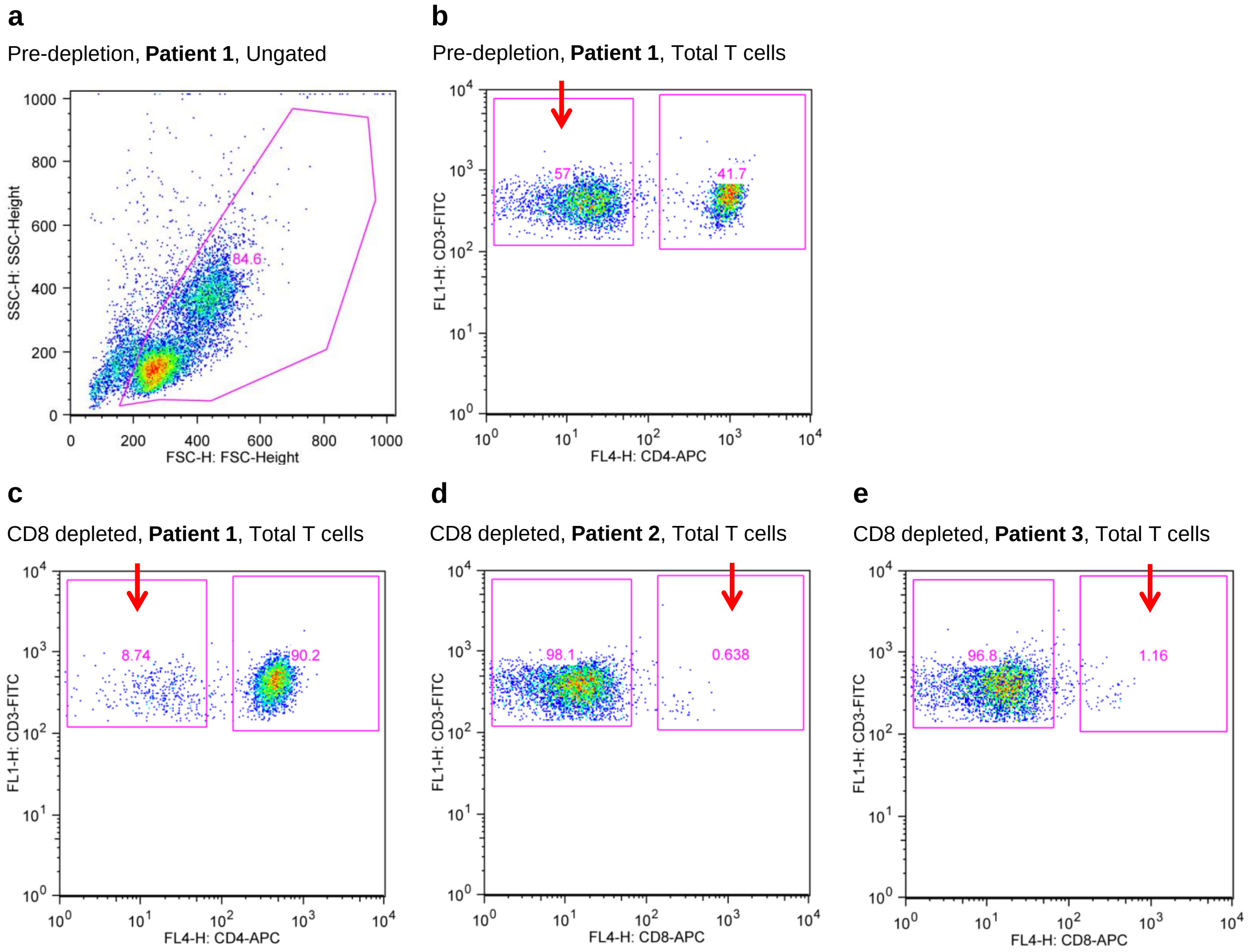

Supplementary Figure S3. CD8⁺ T cell depletion of HIV-infected PBMCs from clinical patients. PBMCs from 3 clinical patients were depleted of $CD8^+$ T cells as detailed in Methods and analyzed by flow cytometry. (**a**) Representative data of T cells within the PBMC population before depletion (circled) viewed by forward and side scatter plot. (**b**) Representative data from patient 1 of the $CD8^+$ T cell population (arrow) in the T cell total deduced from immunostaining with both anti-$CD3^+$ (FITC) and anti-$CD4^+$ (APC) antibodies prior to depletion. (**c-e**) Results of depleting $CD8^+$ T cells from the PBMCs of 3 patients. The $CD8^+$ T cell population remaining in each patient's PBMCs after depletion are highlighted (arrows). This data was deduced from immunostaining cells with an anti-$CD3^+$ (FITC) antibody for total T cells followed by probing with either (**c**) an anti-$CD4^+$ (APC) or (**d-e**) anti-$CD8^+$ (APC) antibody to detect the undesired or desired $CD4^+$ and depleted $CD8^+$ T cell fractions, respectively.

Supplementary Figure S4

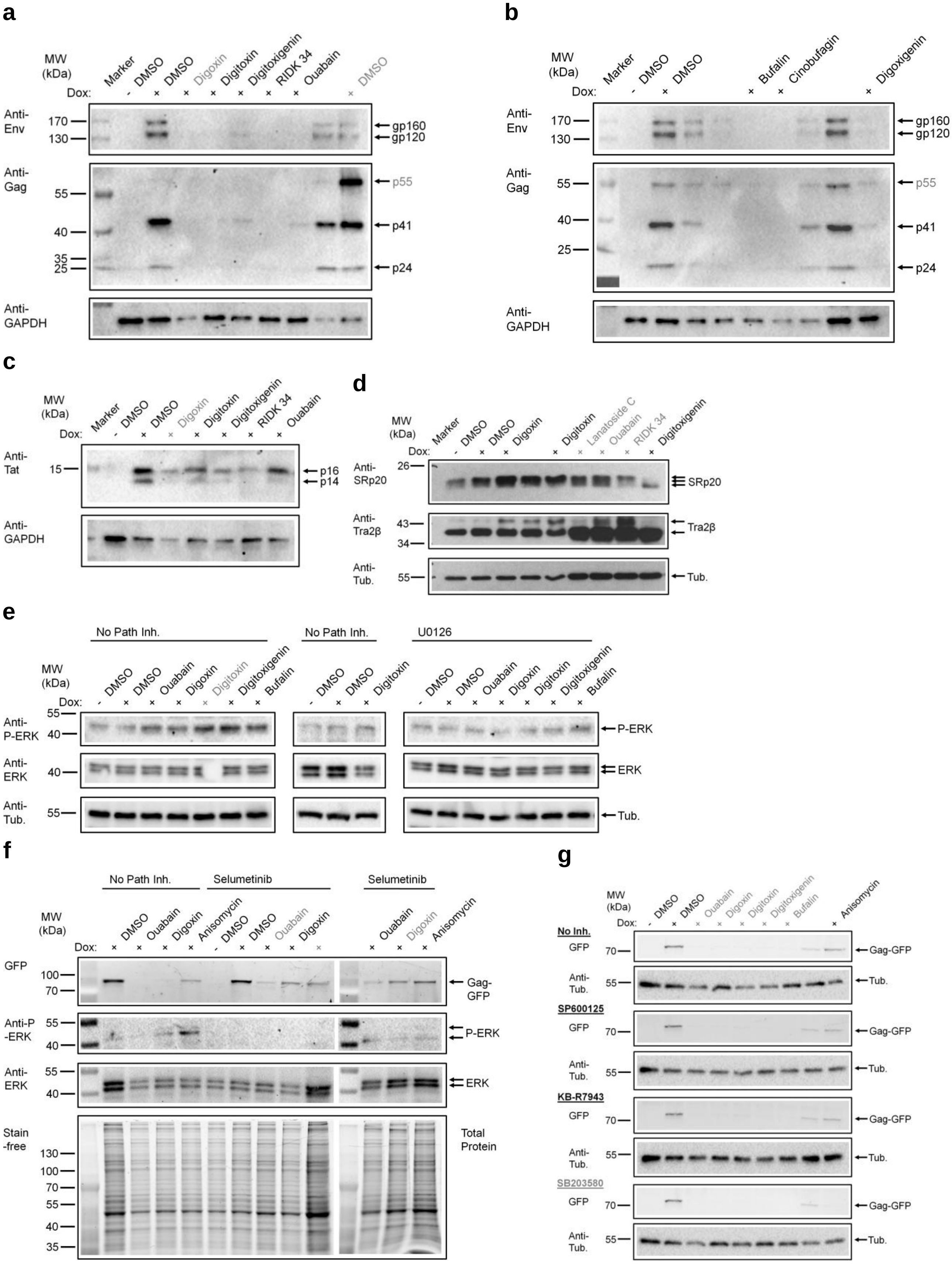

Supplementary Figure S4. Immunoblot/gels used for representative data figures. Lanes from continuous unexcised blot/gels were cropped and rearranged for **Figures 3a** (**a-b**), **3d** (**c**), and **4d** (**d**) and **Supplementary Figures S9c** (**e**)**, S10c** (**f**), and **S12c** (**g**). Samples in gray were not displayed in figures.

Supplementary Figure S5

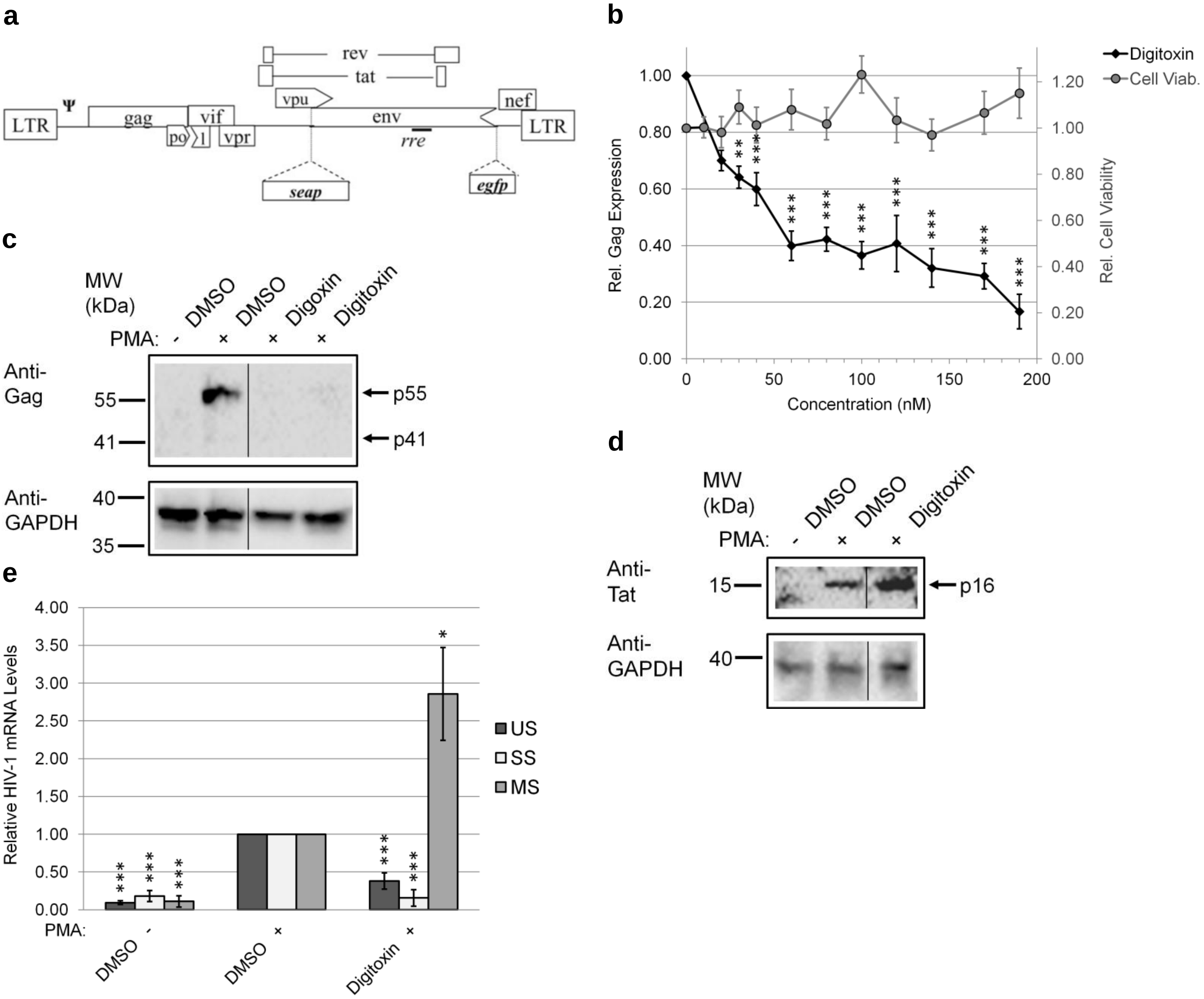

Supplementary Figure S5. Effect of CSs on HIV-1 RNA processing in HIV-1-infected CD4⁺ T cells. 24ST1NLESG cells were treated with 190 nM (or indicated concentrations) of digitoxin, 320 nM of digoxin, or DMSO for 4 h and viral gene expression was induced by addition of PMA. After 24 h, media or cells were harvested for analyses. (**a**) Schematic diagram of the 24ST1NLESG cell line containing a modified HIV-1 proviral genome, NLE⁻S-G (a *pNL4-3* strain), stably integrated into a human acute lymphoblastic lymphoma T-cell line, SUPT1. (**b**) Dose-dependent inhibition of HIV-1 gene expression after digitoxin treatment of 24ST1NLESG cells (n ≥ 4, mean, s.e.m.). HIV-1 Gag expression was assayed by $p24^{CA}$ ELISA of cell supernatants (black circles) and viability of cells assayed by XTT (gray circles, adjacent y-axis) as previously described for digoxin. (**c-d**) HIV-1 Gag but not p16 Tat are altered by digitoxin treatment of T cells (representative of n ≥ 3). Cell lysates were analyzed by western blot with antibodies specific for (**c**) HIV-1 structural protein, Gag, and (**d**) viral regulatory factor, Tat. GAPDH served as internal loading control. Lanes were cropped and assembled from the same blot. (**e**) Digitoxin induces oversplicing of HIV-1 RNAs in T cells (n ≥ 5, mean, s.e.m.). Total mRNA was extracted from treated cells, reverse transcribed, analyzed by qRT-PCR, and the abundance of each HIV-1 RNA class displayed relative to DMSO (+) control as described in **Figures 4a-b**.

Supplementary Figure S6

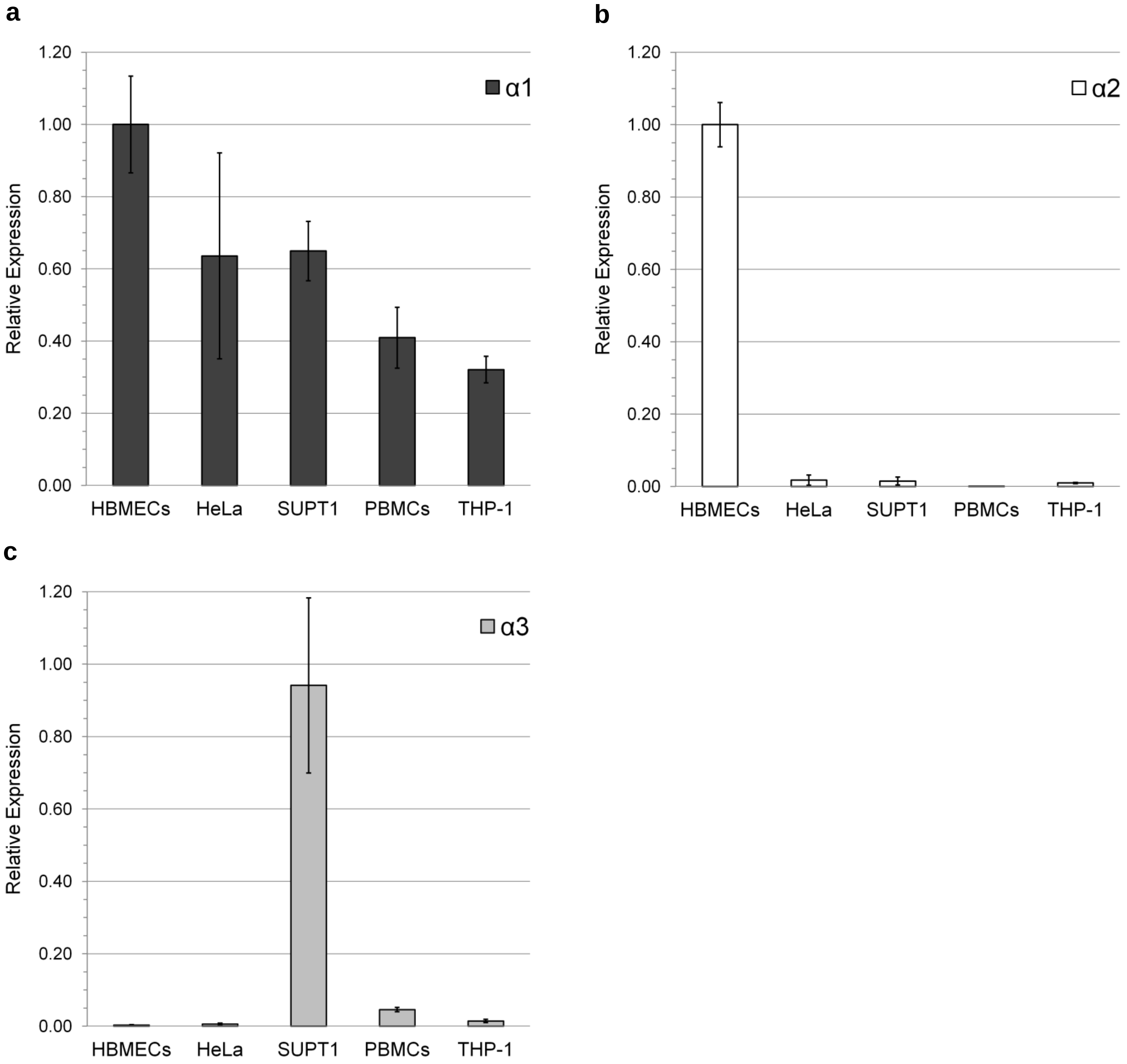

Supplementary Figure S6. Expression of different NKA α-subunit isoforms across various cell types. Total mRNA was isolated from several human cell types, reverse transcribed, and analyzed by qRT-PCR, with data normalized to β-actin, as described in Methods. The expression of α1 and 2 subunits (**a-b**) are shown relative to human brain microvessel endothelial cells (HBMECs) while α3 (**c**) are displayed relative to a T lymphoblast cell line, SUPT1. cDNAs were assayed from the following: HBMECs using a sample run twice in triplicate/quadruplicate, HeLa [rtTA-HIV(Gag-GFP) or rtTA-HIV-Δ*Mls*] cell lines using 1-2 different samples from 3-4 experiments, SUPT1 cell line (24ST1NLESG) using 2 different samples from 5-7 experiments, PHA-L activated PBMCs using a duplicated sample run in duplicate, and human acute monocytic cell line (THP-1, differentiated into macrophages by PMA) using a sample run in quadruplicate. Cells with a relative expression of <0.05 for a given α subunit were considered as background or confirmed negative.

Supplementary Figure S7

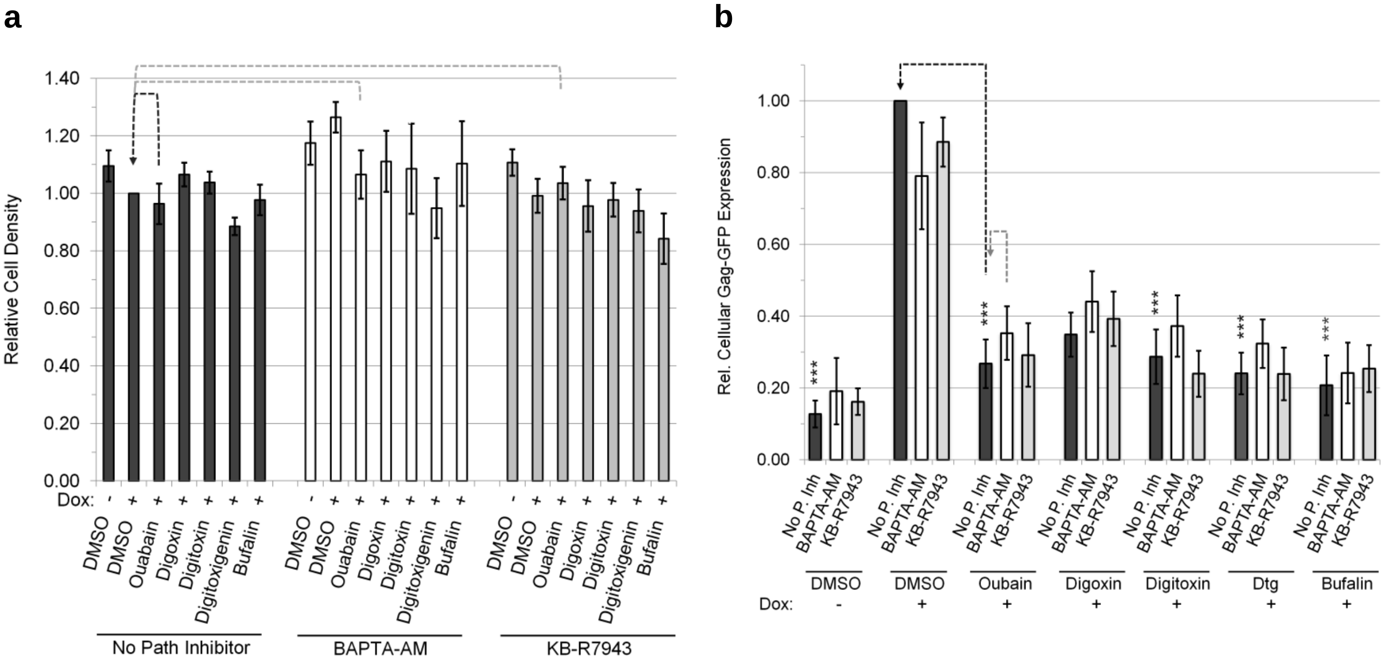

Supplementary Figure S7. CSs inhibit HIV-1 gene expression in a mode independent of changes in $[Ca^{2+}]_i$. HeLa rtTA-HIV(Gag-GFP) cells were pre-treated or untreated (no pathway inhibitor, black) with an intracellular Ca^{2+} chelator (5 µM BAPTA-AM, white) or NCX Ca^{2+}-influx inhibitor (5 µM KB-R7943, gray) for ~2 h prior to treatment with ~IC_{80}s of CSs or DMSO, and Dox induced (as described in **Figs. 5d-f**). To determine the signaling pathway used by a CS, cells were monitored for rescue of Gag-GFP expression by scanning for GFP fluorescence. (**a**) Treated cells demonstrate limited effects on cell density (n ≥ 4 and ≥ 3, resp., mean, s.e.m.). Methylene blue staining was used to detect the density of cells in plates. (**b**) Changes in Ca^{2+} flux are not responsible for CS suppression of HIV-1 gene expression. Graph quantifying Gag-GFP expression in treated cells (n ≥ 3 and ≥ 9, resp., mean, s.e.m.). Statistical comparisons were performed as illustrated (black or grayed dashed lines) and described in Methods. Inhibitor activity and results described in (**b**) were confirmed by Fura Red™ AM (**Fig. 5d**) and SDS-PAGE analysis of Gag-GFP expression (**Figs. 5e-f**).

Supplementary Figure S8

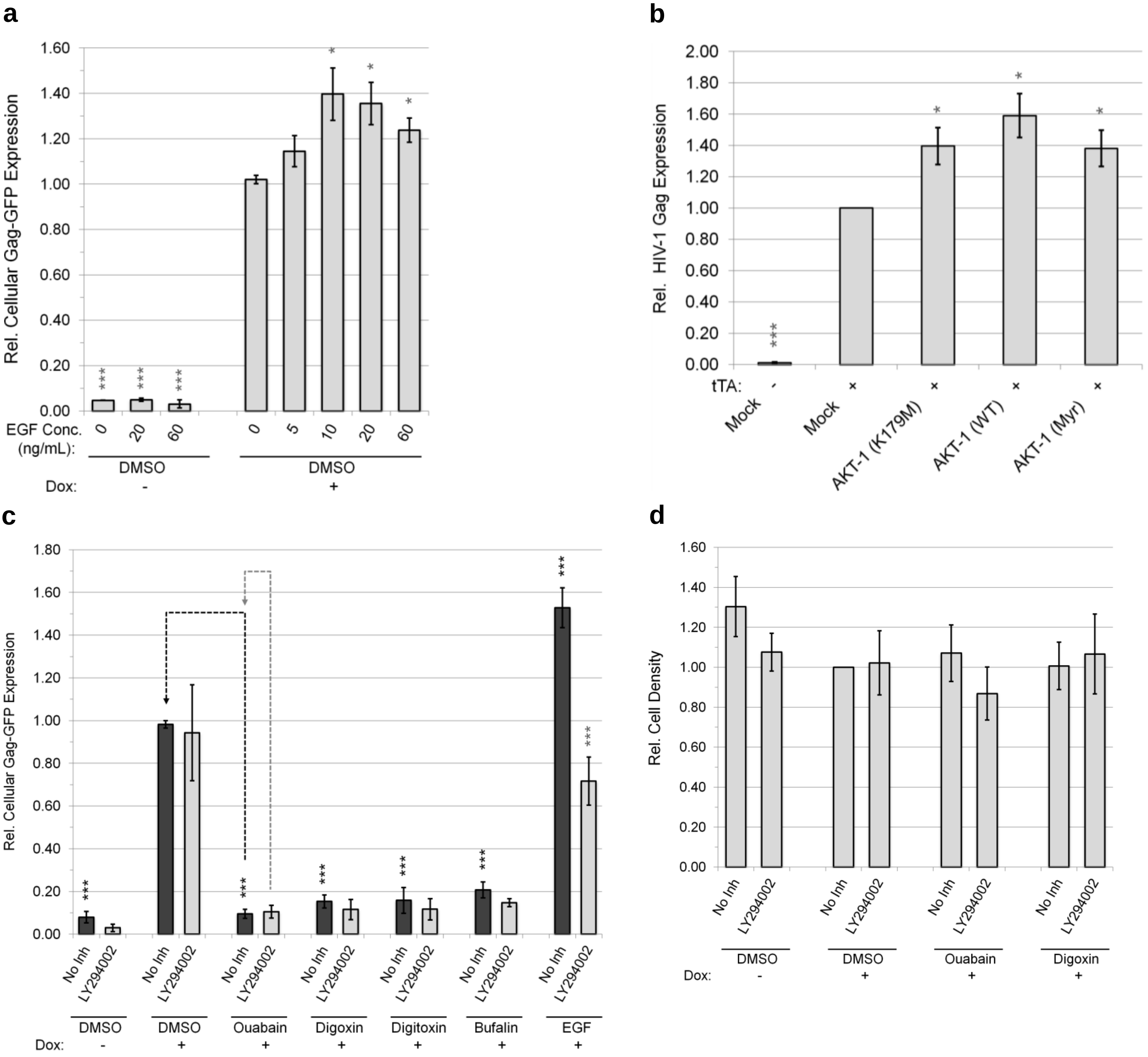

Supplementary Figure S8. PI3K-AKT signaling plays little to no role in the suppression of HIV-1 gene expression by CSs. (**a, c-d**) Signaling pathways activated by CSs to inhibit HIV-1 gene expression were determined by monitoring for rescue of Gag-GFP expression via scanning for fluorescence in HeLa rtTA-HIV(Gag-GFP) cells after pre-treatment with/without (**a**) 0-60 ng/mL of EGF for ~1 h (n = 3, mean, s.e.m.) or (**c-d**) 10 μM of LY294002 for ~2 h (n ≥ 3-8, mean, s.e.m.), treatment with ~IC_{80}s of CSs or DMSO, and Dox induction for 20 h. Results in (**c**) were confirmed by $p24^{CA}$ ELISA in **Figure 5g** and the inhibitory activity of LY294002 on PI3K activity was confirmed by suppression of EGF induction of viral expression. (**d**) Density of cells treated in (**c** and **Fig. 5g**) were determined by methylene blue stain (n ≥ 3, mean, s.e.m.). (**b**) Expression of various forms of AKT-1 in cells demonstrate little effect on HIV-1 gene expression. HeLa rtTA-HIV-Δ*Mls* cells were transfected with HA-tagged AKT-1 plasmid [kinase dead (K179M), wild-type (WT), or constituitively-active myristoylated (Myr)] or mock plasmid and HIV-1 gene expression activated by co-transfection with tTA (+). After 48 h, HIV-1 Gag expression in cell lysates (30 μg) were quantified by $p24^{CA}$ ELISA and displayed relative to mock (+) transfected cells (n ≥ 3, mean, s.e.m.). Statistical analyses were performed as illustrated (black or grayed dashed lines) or to DMSO (+, with 0 ng/mL of EGF) or Mock (+).

Supplementary Figure S9

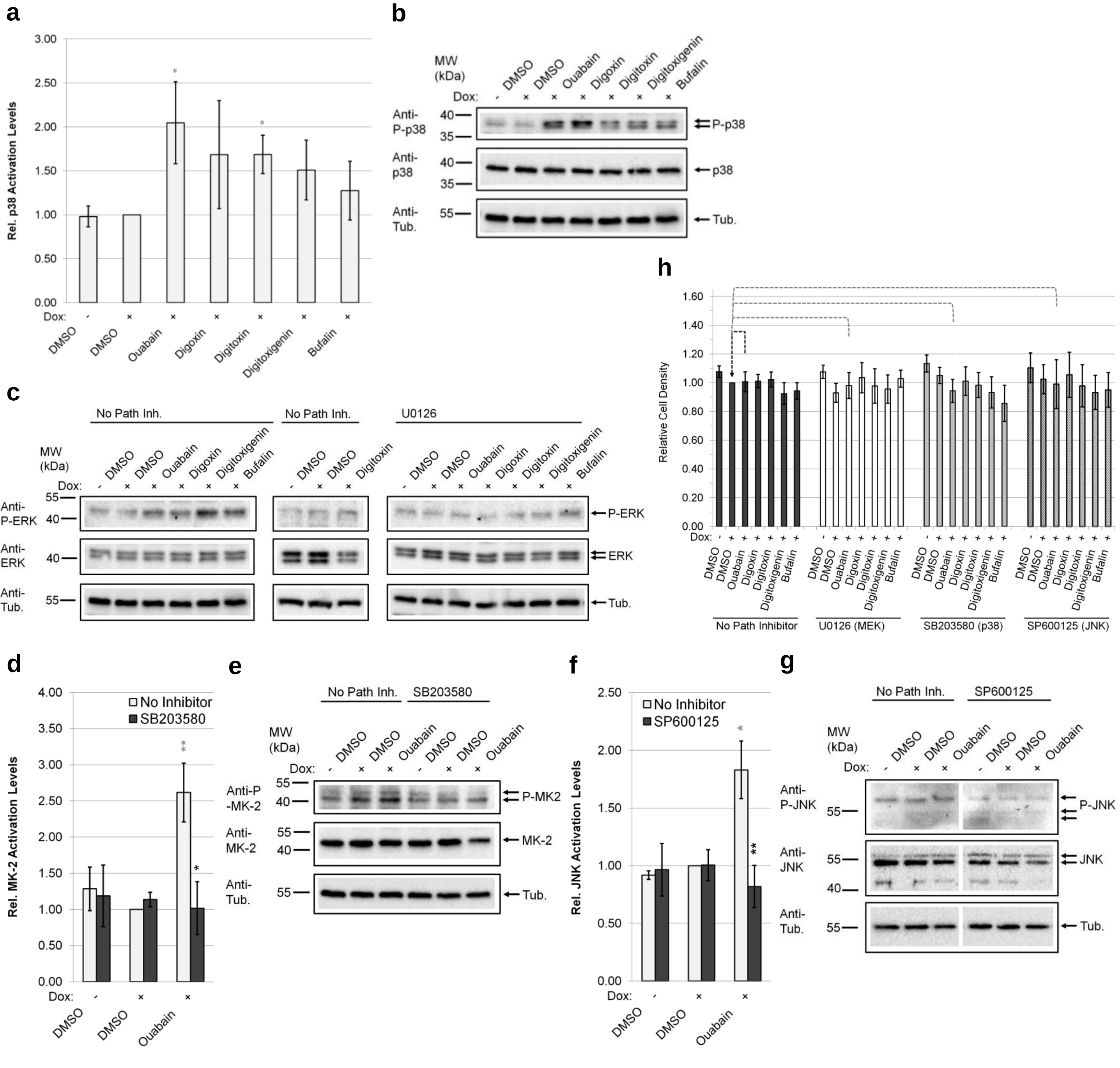

Supplementary Figure S9. Activation of various MAPKs by CSs can be blocked by specific inhibitors. HeLa rtTA-HIV(Gag-GFP) cells were pre-treated with/without a pathway inhibitor overnight (~15 h), treated with ~IC_{80}s of CSs/ouabain or DMSO, Dox induced, and analyzed as described in **Figure 6**. Activation levels (phospho/total protein) were determined by specific antibodies for phospho- and total-MAP/MAPK protein and tubulin, which served as internal loading control and for normalization of these data. Results are displayed relative to DMSO (+) control. A MEK1/2 (12 µM U0126), p38α/β/β2 (15 µM SB203580), or JNK1/2/3 (1.25 µM SP600125) inhibitor (black) or no inhibitor (white) were used as indicated. (**a, d, f**) Graphs quantifying the cellular activation levels of MAPKs (mean, s.e.m.): p38 (**a**, n ≥ 4), ERK1/2 (shown in **Fig. 6a**), MK-2 (**d**, downstream target of p38, n ≥ 3-4), and JNK1/2/3 (**f**, n ≥ 3-6). (**b**, **c**, **e**, **g**) Representative immunoblots for (**a**, **d**, **f**). Lanes in (**c**) were cropped and assembled from the same blot per box per column from **Supplementary Figure S4e** and, for (**g**), from continuous parts assembled from the same blot. (**h**) Combinations of pathway inhibitor and CSs applied demonstrate little/no change in cell density as assayed by methlene blue stain (n ≥ 3-6, mean, s.e.m.).

Supplementary Figure S10

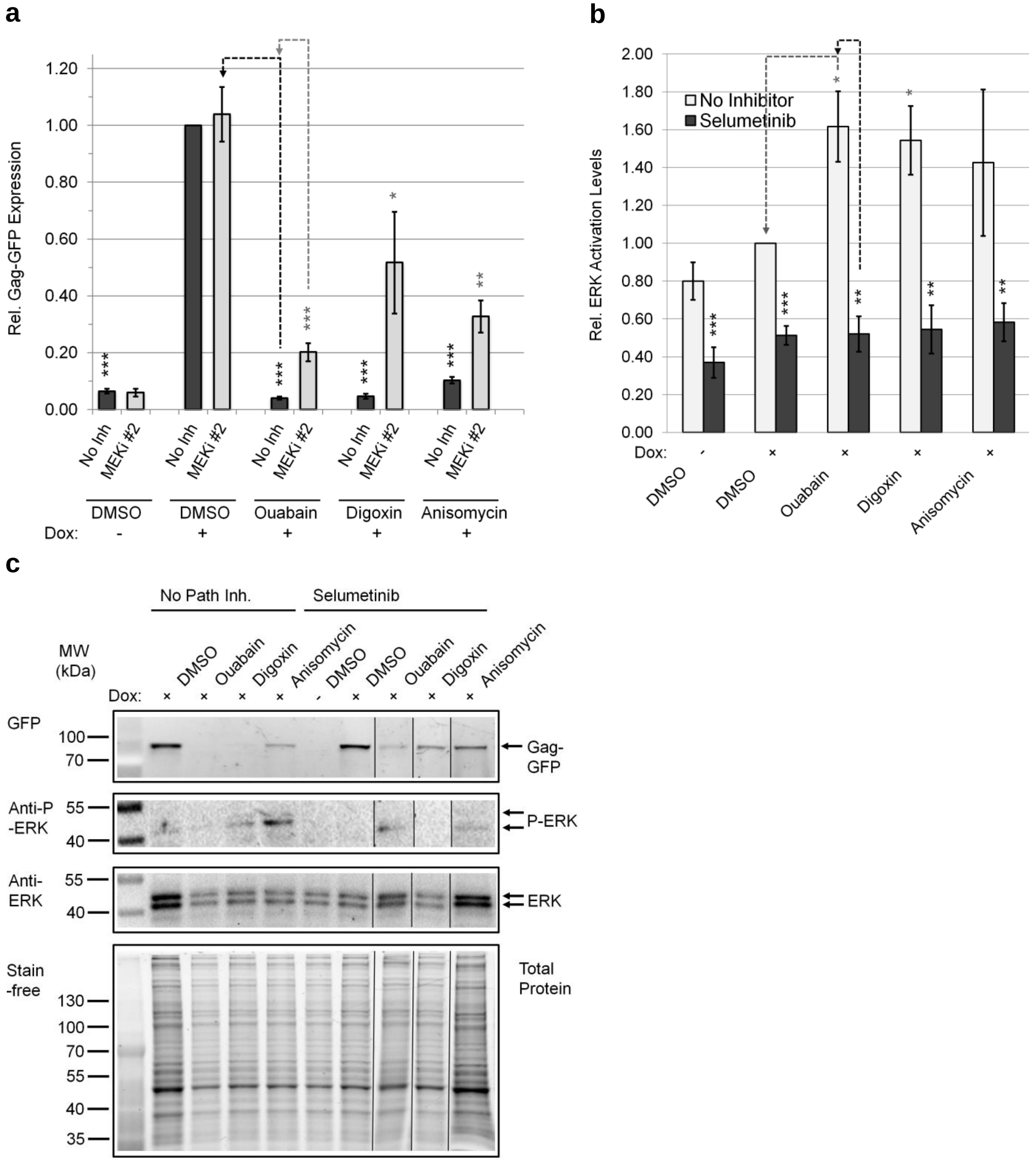

Supplementary Figure S10. CSs and anisomycin inhibit HIV-1 gene expression through MEK1/2-ERK1/2 signaling. HeLa rtTA-HIV(Gag-GFP) cells were pre-treated with/without a second MEK1/2 inhibitor, Selumetinib (MEKi #2, 5.5 µM), for ~15 h, treated with 27 nM Ouabain, 70 nM Digoxin, 220 nM of Anisomycin, or DMSO, Dox induced, and analyzed as described in **Figure 6**. Lysates of cells (~20 µg) were resolved on reducing SDS-PAGE to quantify rescue of HIV-1 Gag-GFP expression via detection of GFP fluorescence and gels were immunoblotted by specific antibodies for phospho-ERK1/2 and total-ERK1/2 to determine the levels of ERK activation. Stain-free™ gel staining was used to monitor total protein load and for normalization of these data. (**a**) Graph quantitating relative Gag-GFP expression in treated cells (n ≥ 3, mean, s.e.m.). (**b**) Graph showing relative ERK activation in treated cells (n ≥ 3, mean, s.e.m.). (**c**) Representative gels and immunoblots of (**a-b**). Results were shown relative to DMSO (+). Statistical comparisons were performed as illustrated (black or grayed dashed lines). Blots in (c) were cropped and assembled from **Supplementary Figure S4f**.

Supplementary Figure S11

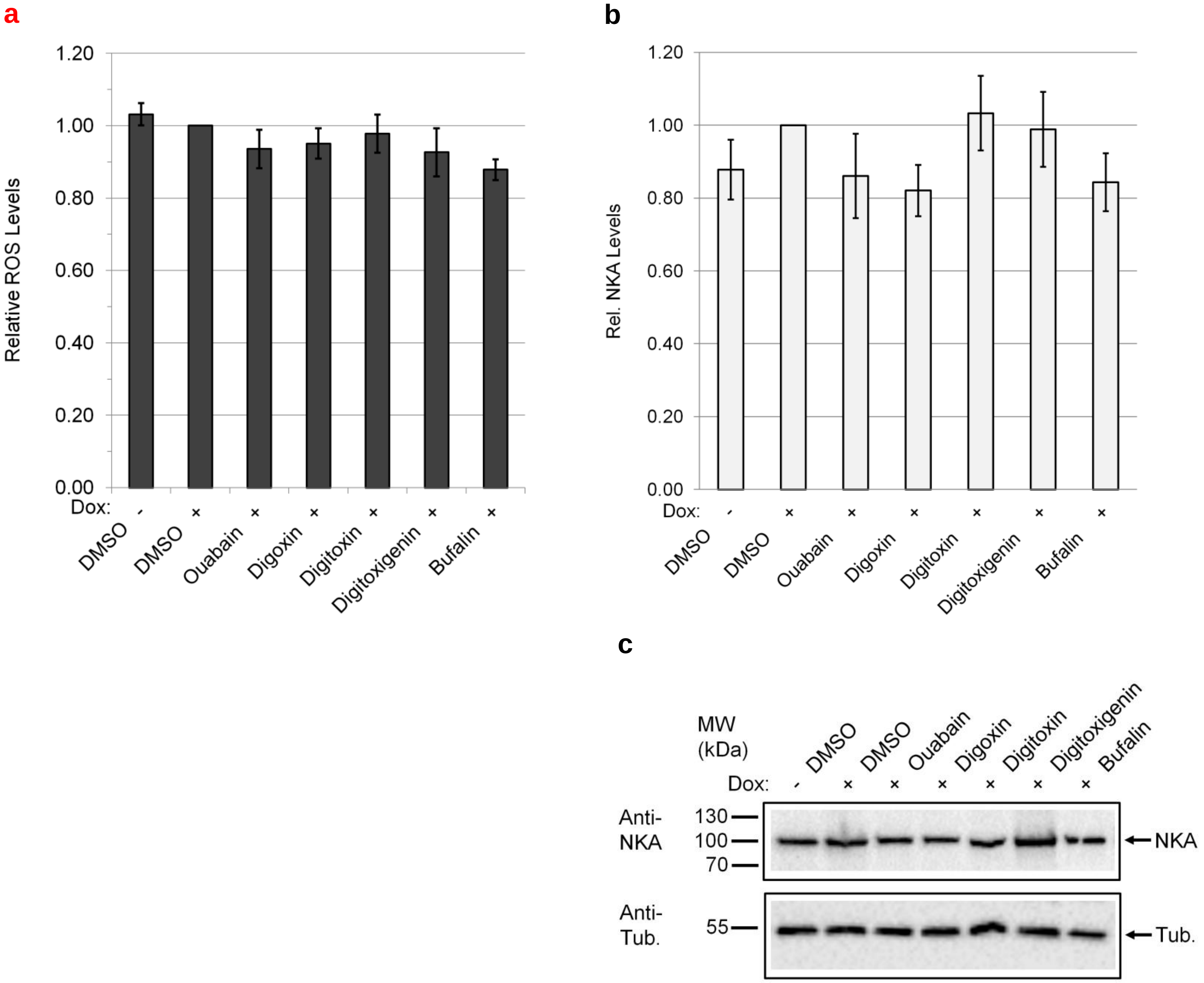

Supplementary Figure S11. Effect of CSs on ROS production and NKA levels in cells. HeLa rtTA-HIV(Gag-GFP) cells were treated with ~IC_{80}s of CSs or DMSO for 4 h and viral gene expression induced (+) by Dox for 20 h. (**a**) Intracellular concentrations of ROS are unaltered upon exposure of cells to CSs ($n \geq 3$, mean, s.e.m.). ROS levels in cells were monitored by CellROX® Deep Red labeling (as described in Methods) and results graphed. (**b-c**) CS treatment of cells cause little to no change in levels of NKA ($n \geq 3$, mean, s.e.m.). The amount of NKA in cell lysates were monitored by western blot using specific antibodies to the NKA while α-tubulin blots served as internal loading control and for normalization of this data. (**b**) Graph quantitating the levels of NKA. (**c**) Representative immunoblot of (**b**). Results are displayed relative to DMSO (+) control. Statistical analyses (**a-b**) were performed as described in Methods.

Supplementary Figure S12

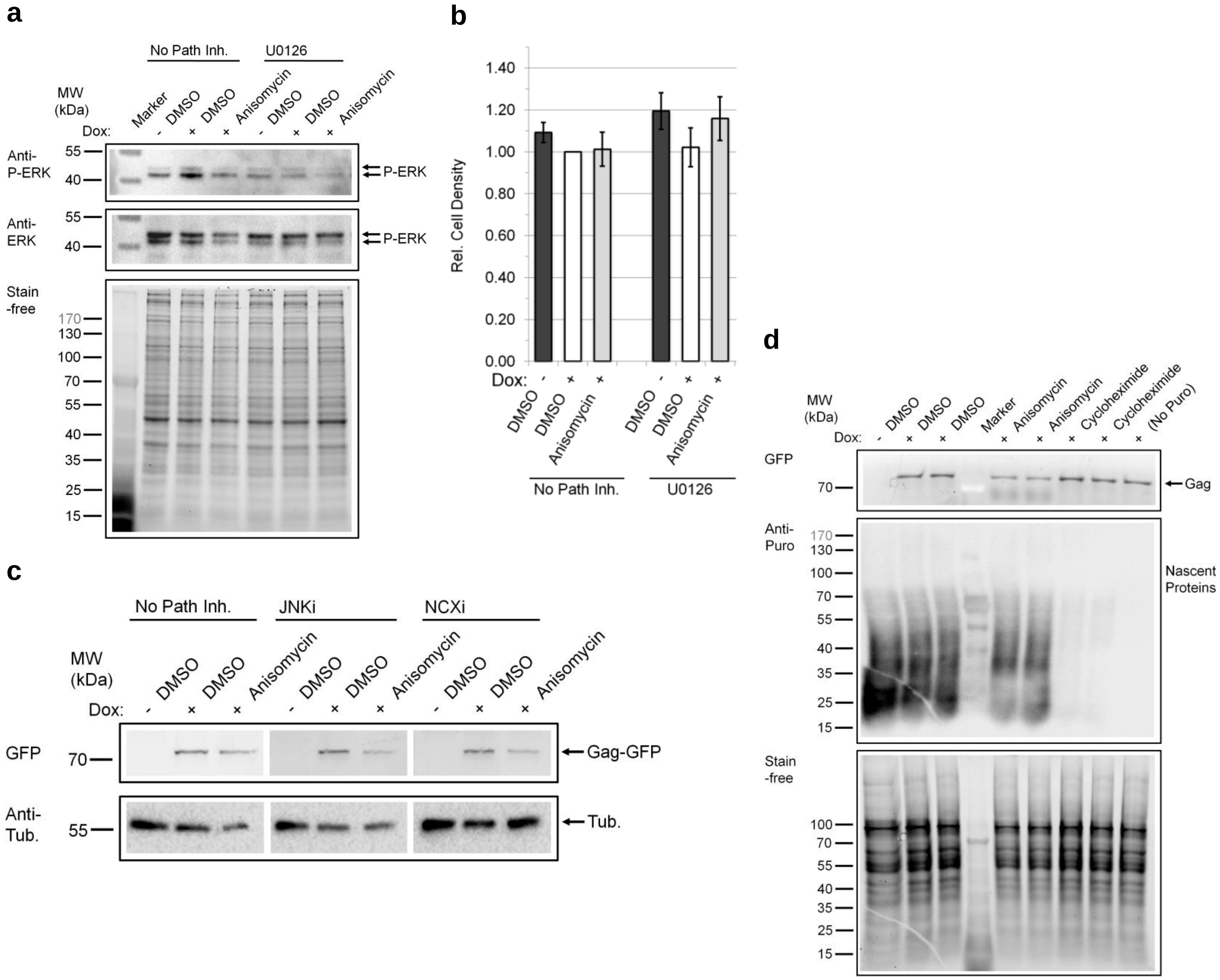

Supplementary Fig. S12. Role of anisomycin signals on the inhibition of HIV-1 gene expression and protein synthesis. HeLa rtTA-HIV(Gag-GFP) cells were pre-treated with either a MEK1/2 (12 µM U0126) or JNK1/2/3 (1.25 µM SP600125, JNKi) pathway inhibitor overnight (~15 h), a NCX inhibitor (5 µM KB-R7943, NCXi) for ~2 h, or DMSO overnight or ~ 2h, treated with anisomycin (220 nM) or DMSO for ~4 h, Dox induced for 20 h, and analyzed per **Figure 7a-e**. A translation inhibitor, cycloheximide (100 µg/mL), was added as a control. The cellular signal used by anisomycin to inhibit HIV-1 gene expression was determined by assaying for rescue of Gag-GFP expression/fluorescence in cell lysates (~35 µg) resolved on SDS-PAGE. Effects on ERK1/2 activation were quantified by immunoblots of phospho- and total-ERK1/2 and newly synthesized proteins were quantitated by SUnSET by immunoblots of puromycin-labeled proteins. The activity of each pathway inhibitor was confirmed and run in parallel with experiments in **Figure 6a**, **Supplementary Figures S9c-g,** and **Figure 5d**. Stain-free™ labeled total protein or tubulin were used as internal loading controls and for normalization of these data. Results are displayed relative to DMSO (+) control. (**a**) Representative immunoblot displaying ERK1/2 activation levels in treated cells (representative of n ≥ 4-5). Graph of results in (**a**) are found in **Figure 7d**. (**b**) Graph of cell density after drug treatments assayed by methylene blue stain (n ≥ 4-5, mean, s.e.m.). (**c**) Representative gel of Gag-GFP expression in treated cells (representative of n ≥ 3). Lanes in (**c**) were cropped and assembled from same gel/blots from **Supplementary Figure S4g**. (**d**) Representative gel of Gag-GFP expression and blot of nascent synthesized proteins in cells treated with anisomycin in duplicate (representative of n ≥ 3). The translation inhibitor cycloheximide was added as a control.

Supplementary Figure S13

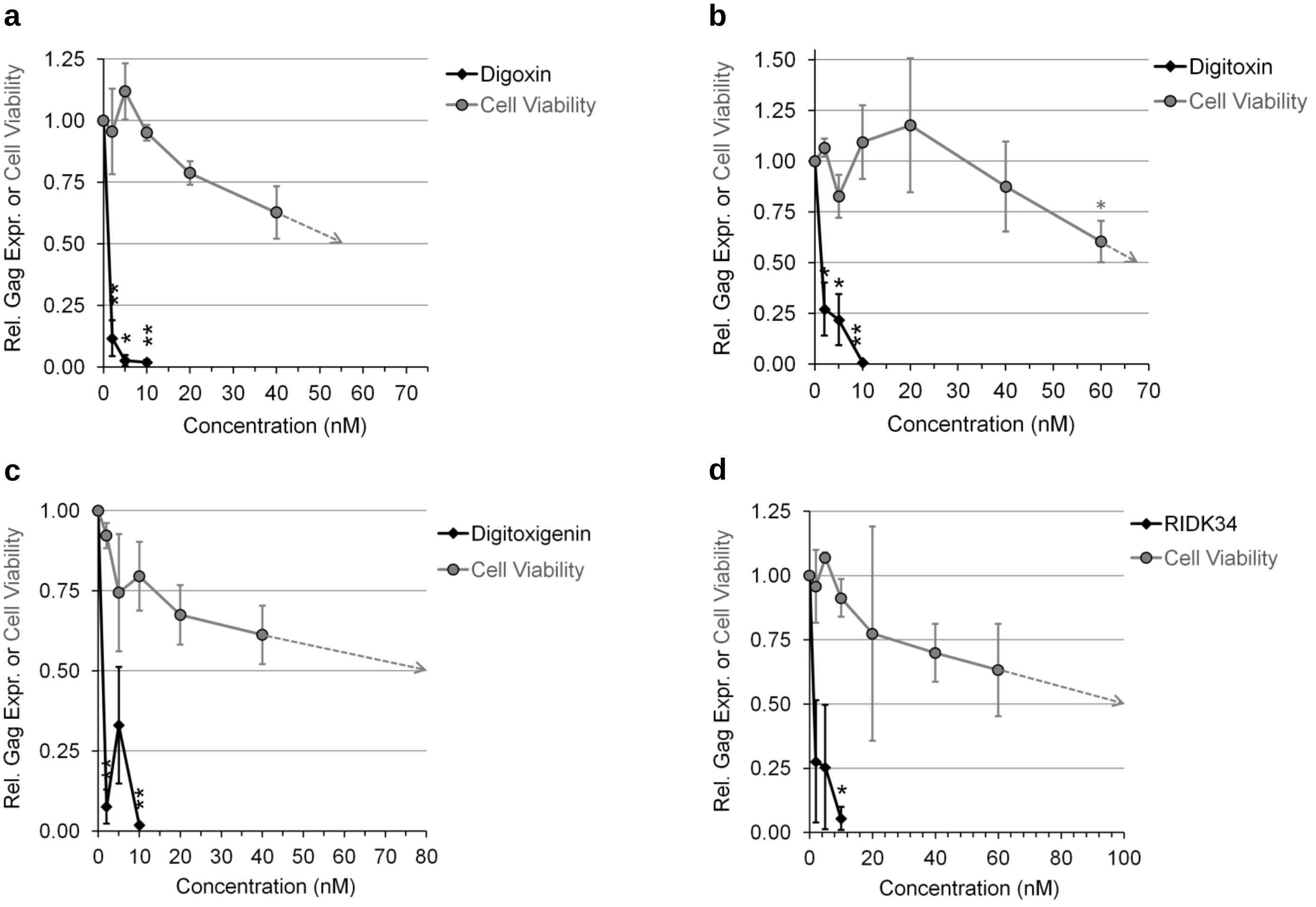

Supplementary Figure S13. Estimation of the approximate *ex vivo* therapeutic indices of several CSs. *Ex vivo* TIs were calculated from CC_{50}/IC_{50} from day 14 of culture of HIV-infected patient PBMCs treated with CSs. CC_{50}s were estimated/extrapolated from data points by their trends (dashed arrows) from XTT assays of cell viability (gray circles) and their inferred near maximal IC_{50}s estimated from results on HIV-1 $p24^{CA}$ (Gag) production (black circles) from **Figures 2f-h** and some data points published on digoxin [Wong *et al.* (2013). *PLoS Pathog* **9**(3):e1003241]. This data is summarized in **Supplementary Table 1.**

.

Supplementary Figure S14

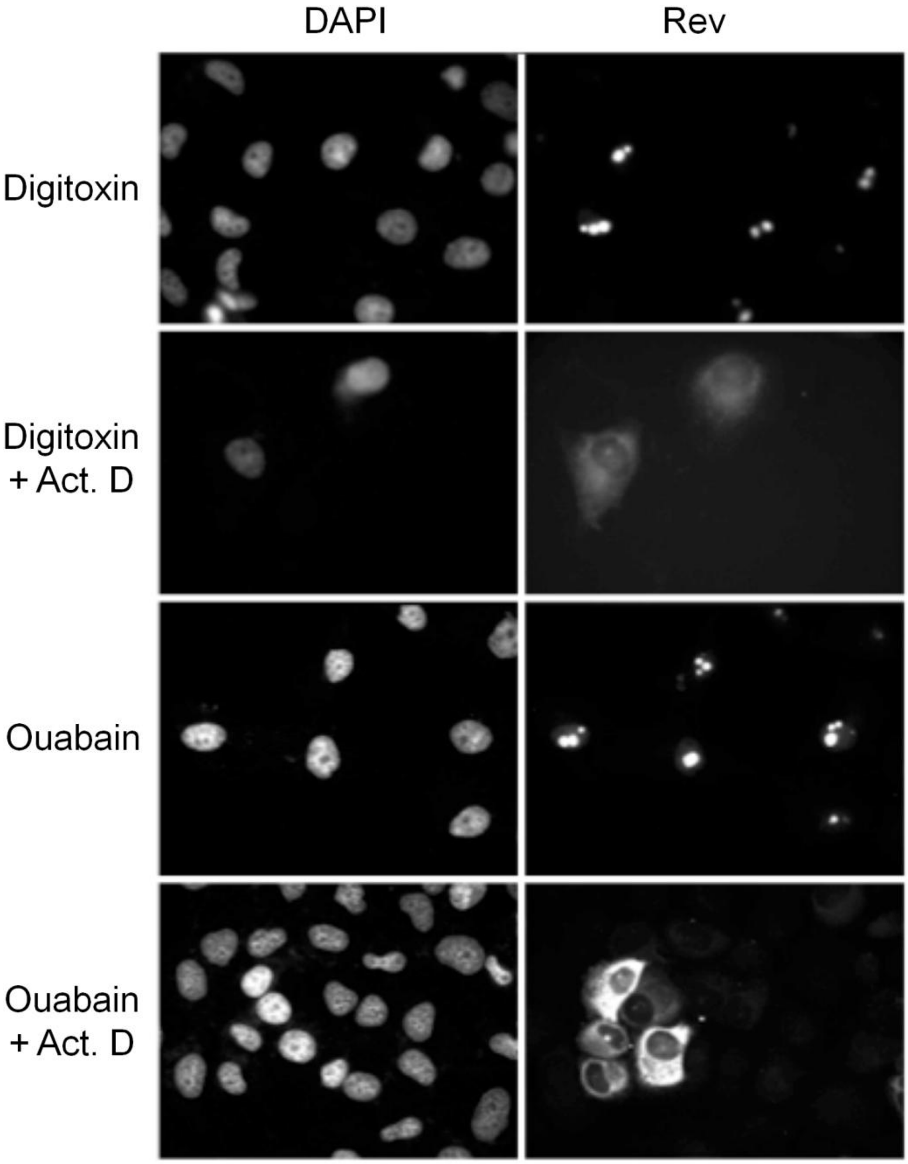

Supplementary Figure S14. CSs do not alter the subcellular localization or transport function of HIV-1 Rev. HeLa cells stably transfected with a constitutively active Rev were treated with CS (50 nM of digitoxin or ouabain) or with DMSO (control, not shown) overnight and observed in the presence (+) or absence of 4 mg/mL Act. D (reported to cause cytoplasmic localization of Rev) added 2 hours prior to harvest. Cells were fixed, permeabilized, and Rev was immunolocalized by rabbit anti-Rev antibody and FITC/Cy5-conjugated anti-rabbit IgG antibodies. Cells were stained with DAPI to detect nuclei. Images were acquired at 400x magnification. Results are representative of n ≥ 2. Experimental methods were performed as previously described in Wong *et al.* (2013). *Nucleic Acids Res.* **41**(20), 9471–9483.
